# Enhancing the weed segmentation in diverse crop fields using computationally effective concatenated attention U-Net with convolutional block attention module

**DOI:** 10.1038/s41598-025-31285-7

**Published:** 2025-12-16

**Authors:** R. Arumuga Arun, S. Umamaheswari, Islabudeen Mohamed Meerasha, B. Mohankumar

**Affiliations:** 1 School of Computer Science and Engineering, Vellore Institute of Technology, Vellore, Tamil Nadu India; 2 Department of Information Technology, Anna University-MIT Campus, Chennai, Tamil Nadu India

**Keywords:** Computer vision, Deep learning, Convolutional neural network, Semantic segmentation, Patterning, Plant physiology

## Abstract

Weeds are one of the primary factors that reduce crop productivity by competing for nutrients and water, causing the plant to lose weight and resulting in reduced grain yield. Traditional agricultural practices often rely on uniform herbicide application, which can contaminate soil and raise costs. On agricultural land, selective weed treatment are an efficient and cost-effective way to control weeds that require a deep learning-based crop and weed segmentation system. Many existing crop and weed segmentation research works focus on achieving precise crop and weed segmentation results, rather than building lightweight models to deploy on edge devices. To attain this, we develop an effective and efficient convolutional neural network, namely the Concatenated Attention U-Net with Convolutional Block Attention Module (CAUC). By integrating Linear Concatenated Blocks (LCB), Attention Gate (AG) connections, and Convolutional Block Attention Module (CBAM), the proposed model efficiently utilizes feature maps among its architectural components to achieve superior performance. Depth-wise convolution layers and 1 × 1 convolution layers in LCBs reduce computational complexity. To enable the proposed model to identify the weed portions in multiple crop fields, we integrated three datasets in this research work, namely the Crop/Weed Field Image Dataset (CWFID), Sugar Beet, and Sunflower datasets. Experimental results on carrot, sugar beet, and sunflower crop datasets demonstrate high Accuracy (99.09%), MIoU (81.02%), and F1-score (99.06%), with a modest model size (5.6 MB) and computational parameters (0.377 million). We developed a lightweight computer vision application (13.7 MB) to demonstrate the model’s efficacy on low-computational devices.

## Introduction

An increasing population requires India to accelerate the production of crops. Weeds are a big obstacle to higher yields since they complicate farm operations as well as damage rural economies. Because they are competitive, tenacious, and non-edible plants, the weeds compete badly with crops for vital resources such as sunlight, nutrients, water, as well as space. Crop standards as well as farm productivity are both decreased due to this competition. India will be able to significantly increase crop yields as well as food security by addressing these problems as well as implementing effective weed control measures.

Although weeds are randomly spread across the fields, customary agriculture treats the whole field equally to the infested part to address the issues created by the weeds. Application of herbicides, beyond some specific limit, may also pollute the land as well as the soil and increase the cost of cultivation. Thus, an efficient crop-weed detection system must be developed to eliminate weeds selectively and reduce herbicide usage.

A few shortcomings of approaches based on machine learning include the requirement for feature engineering before training and the requirement for sizable, manually created, or well-structured datasets^1^. On the other hand, deep learning automatically extracts features during training, if sufficient data is available, eliminating the need for human feature engineering. Deep learning algorithms are excellent at handling complicated tasks like object detection, image recognition, image classification, and natural language processing than typical machine learning models^2,3,54^.

The main use of a Convolutional Neural Network (CNN), a deep learning model, is the interpretation of visual input, including images and videos. CNNs can create object detection models to find and identify many things in an image and classification models to classify images by automatically extracting significant characteristics^4,5^. However, identifying and differentiating crop and weed areas in the image at the pixel level is necessary for an efficient crop-weed segmentation system. Traditional classification and object identification models are not up to the challenge.

This is possible by creating a CNN-based segmentation model with pixel-wise labeling, where a pixel is labeled with a particular category^6^. The model separates the input image into different classes, like crops and weeds, to enable accurate detection of weed-infested regions. With pixel-wise labeling, a crop-weed segmentation system facilitates selective weed management, enhancing efficiency in farming operations.

The segmentation models, such as SegNet512^7^, UNet^8^, AgNet^9^, and RRUDC^10^, are utilized for crop-weed segmentation. All these models use the encoder and decoder-based structure, where each encoder block corresponds to a decoder block^11^. Many of the existing crop and weed segmentation works are about single crops in nature. Because weeds and crops found in different crop fields have fewer distinctive features and more similar ones, designing a multi crop weed segmentation model is challenging. Hence, an efficient model is necessary to extract these distinct features among the crops and weeds. It can be achieved with dense connection^12^, residual connection^13,14^, Convolutional Block Attention Module (CBAM)^15^, and Attention Gate connection^16,17^. But, at the same time, building a computationally smaller model is also essential to increase the usability of the model, which was accomplished by including the depth-wise separable convolution and 1 × 1 convolution layers in the proposed model.

## Literature survey

The literature review for this research work is centered on three main points. The first investigates ways of creating computationally effective models without loss of performance. The second looks at current research on crop-weed segmentation. Lastly, the third point reviews attention mechanisms applied in different tasks.

### Building of computationally less complex model

Sun et al. (2023) constructed a light semantic segmentation model, namely RL-DeepLabv3+, to deploy on an unmanned rice harvester for rice lodging detection^18^. The most interesting aspect of this work is the application of depth-wise separable convolution methods in the backbone network to provide efficiency enhancement and a residual network to enhance the utilization of feature maps. Likewise, to solve issues in precisely segmenting ocular areas, Naqvi et al. (2020) proposed Ocular-Net, a combination of residual skip connections’ strengths with the SegNet model^19^.

Jang et al. (2023) suggested FALCON, a compression algorithm to compress the size of CNN models without degrading performance^20^. The algorithm applies depth-wise convolution operations and channel concatenation to provide stability with effective model performance. Hossain et al. (2022) introduced RA-CNN, a two-domain deep learning method for MRI image reconstruction^21^. RA-CNN is based on the UNet architecture and incorporates residual connectivity and an attention mechanism to facilitate improved feature use across architectural components.

Chen et al. (2020) used a soft attention mechanism and residual connections to the UNet model for building the Residual Attention UNet to obtain accurate multi-class segmentation on a dataset of CT images^22^. Rampriya & Suganya (2021) utilized the CBAM module in their RSNet to improve railroad segmentation^23^.

Yang et al. (2019) presented the Residual Dense UNet (RDUN), a semantic segmentation network used in detecting road defects. RDUN combines residual learning and dense connections with the UNet architecture to better extract features^24^. Likewise, Li et al. (2019) presented fire-FRDCNN and mobile-FRD-CNN, which apply full concatenation paths to reuse feature maps and include 1 × 1 convolution layers to lower computational costs^25^.

### Existing crop and weed segmentation works

Carbone et al. (2022) utilized the deep learning model Bonnet for sunflower crop field image segmentation of plants and weeds^26^. With the help of RGB and NIR images, the Bonnet model was trained to segment weeds and crops according to semantic areas. Although the method attained 78.98% IoU in crop-weed segmentation, it was not generalizable across sunflower crops. Fawakherji et al. (2021) investigated some of the most popular encoder-decoder models, such as SegNet, UNet, UNet-ResNet, and Bonnet, for crop-weed segmentation of sugar beet crops based on semantic segmentation methods^27^. To improve the accuracy of segmentation, the authors employed efficient augmentation methods, producing synthetic images by utilizing a conditional GAN (cGAN).

For segmenting the crop and weed segmentations, Hashemi-Beni et al. (2022) investigated various CNN models, namely UNet, SegNet, DepLabV3+, FCN-8s, FCN-16s, and FCN-32s in their research work^28^. These experiments were carried out on two UAS imagery Datasets, the CWFID and the Sugar Cane Orthomosaic datasets, where the DepLabV3+ (84.3%) and FCN-8s (76.62%) outperformed others in terms of overall classification accuracy on the CWFID and Sugar Cane datasets, respectively. The models’ overall classification accuracy could have been more significant, and the proposed approach was single-crop-based.

Nasiri et al. (2022) used ResNet50 as the encoder in their proposed UNet model for crop-weed segmentation^29^. The images taken from the sugar beet agricultural fields are used to train their proposed UNet model, and they attained an accuracy of 96.06% with an IoU value of 84.23%. The proposed model was not, however, made computationally efficient or to handle multiple crops.

Wang et al. (2020) suggested an encoder-decoder model with pixel-wise labeling to segment crop and weed areas in sugar beet fields^1^. For the sake of efficiency, the computational parameters of the model were minimized through depth-wise convolution methods. This method was restricted to the sugar beet dataset. Likewise, Zou et al. (2021) utilized the UNet model for crop-weed segmentation and used efficient augmentation methods to increase the training dataset^30^.

Fawakherji et al. (2019) suggested a deep-learning approach for efficient crop and weed classification of real-world field images^31^. The method employed two sequential CNNs: VGG-UNet for vegetation segmentation and VGG16 to distinguish the crop and weed patches. Nevertheless, the approach is time and computationally costly because of the application of two VGG16 models in a two-stage process.

### Exploring attention mechanisms in diverse applications

To identify image anomalies, Zhang and Tian (2023) developed a Transformer model with mutual attention^32^. Their method enhances the interaction among image regions, allowing for easier identification of anomalies. Mutual attention aids by taking into account relationships among different image features. In virtual reality (VR) applications, Xiao et al. (2024) built a Multi-Scale Spatio-Temporal Attention Network (MSSTANet) to enhance human action recognition (HAR)^33^. The approach overcomes issues in the precise recognition of user actions by extracting both spatial and temporal features from action signals. Liu et al. (2021) presented NHBS-Net in their work^34^, which is a dedicated neural network that is meant to improve the segmentation of ultrasound images of neonatal hip bones. The network has a feature fusion attention mechanism for enhancing the accuracy and robustness of segmentation.

Lin et al. (2021) presented the Efficient Attention Pyramid Transformer (EAPT), an attention-based hierarchical model for image classification, object detection, and semantic segmentation. The innovation of EAPT is its multi-scale attention mechanism, which effectively captures both global context and local dependencies in images^35^. Nazir et al. (2021) proposed ECSU-Net, a neural network that enhances intervertebral disc (IVD) segmentation and classification from CT images^36^. The model addresses spinal analysis difficulties by employing specific modules to optimize accuracy and efficiency.

## Materials and methodology

The primary goal of this work is to design a CNN-based deep model for crop and weed segmentation in farmland with pixel-wise labeling. The model will also label crops and weeds into distinct categories for various fields. The model should correctly identify them while being efficient enough to be executed in low-power devices.

### Data pre-processing

**Fig. 1:**
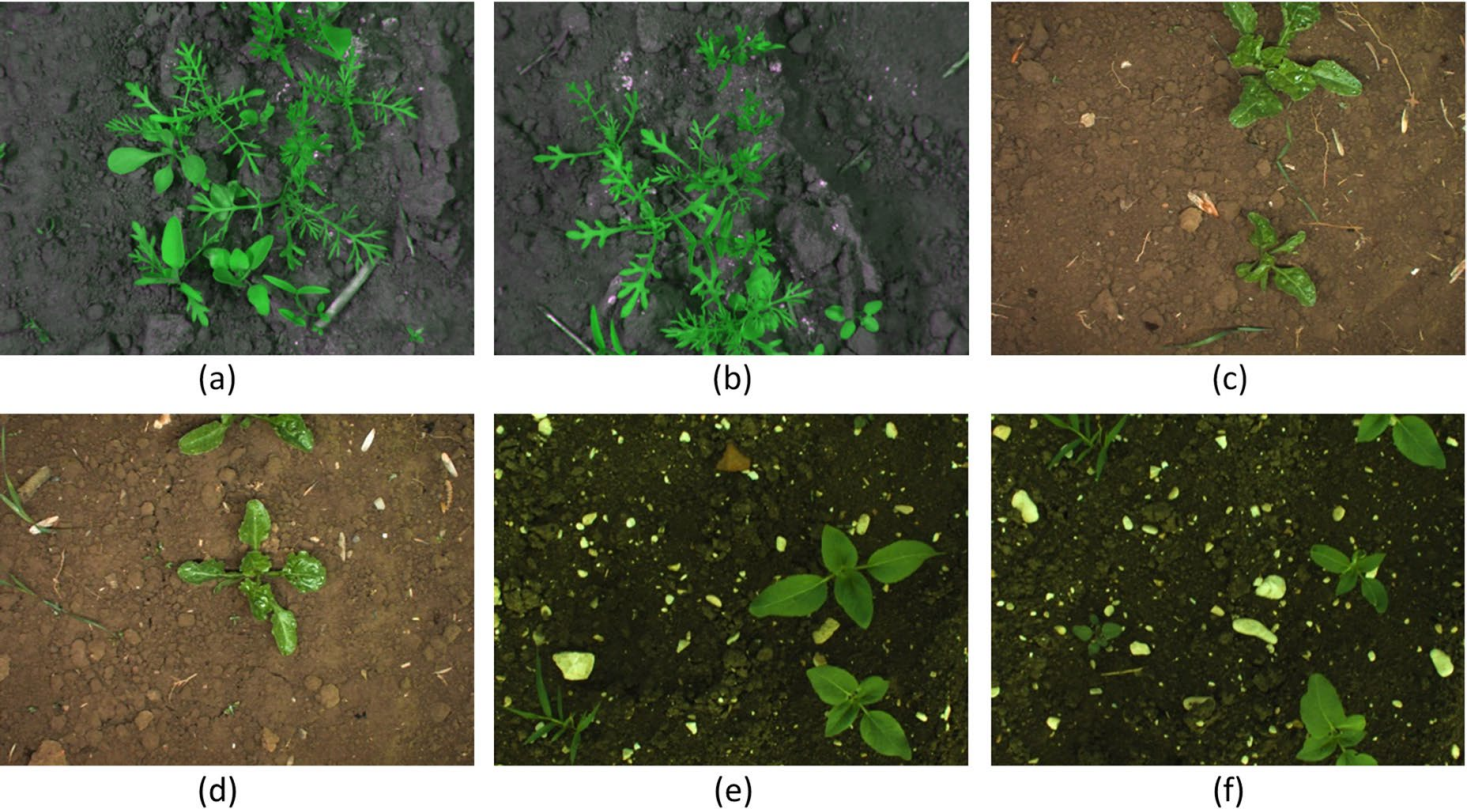
The sample images from the dataset used for training the model. (**a**,**b**) Carrot crop field images, (**c**,**d**) Sugar Beet crop field images, and (**e**,**f**) Sunflower crop field images.

Building a precise deep learning model requires a rich training dataset. Most high-performance models are not successful in actual farms due to their training on lab images. To succeed in the real world, all training images should be from actual farms. For this reason, this study applies only actual agricultural field images. Sample images are presented in Fig. 1.

**Fig. 2:**
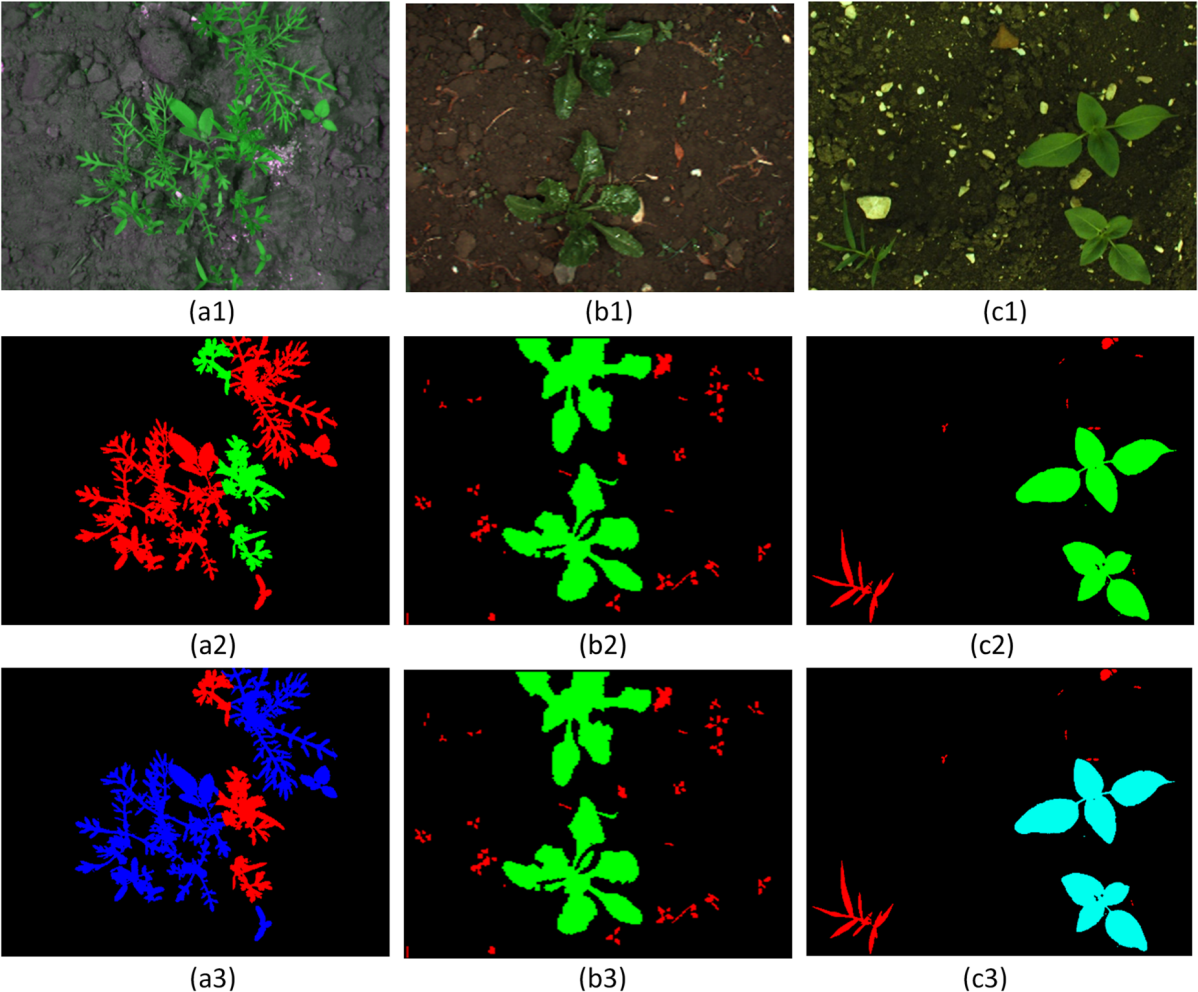
The sample actual field images, target label images, and re-colored target label images of the three datasets. (**a1**,**b1**,**c1**) actual field images (**a2**,**b2**,**c2**) original target label images, and (**a3**,**b3**,**c3**) re-colored target label images of CWFID, Sugar Beet, and Sunflower datasets.

A dataset that comprises various crops and their potential weeds is required to build a multi-crop weed detection model. We have combined three existing datasets to create the necessary dataset for this research, since any such dataset that includes multiple crops and weeds is not currently present. The three datasets utilized are the Crop/Weed Field Image Dataset (CWFID)^37^, the Sugar Beet (SB) dataset^38^, and the Sunflower (SF) Dataset^27^, where the crops are carrot, sugar beet, and sunflower. All these datasets contain actual crop field images and their corresponding target label images. Both of these actual and label images are required for training a vision-based segmentation model since they enable supervised learning. A sample of the actual agricultural field image and the corresponding label image of each dataset is visualized in Fig. 2(a1, b1, c1) and 2(a2, b2, c2), respectively.

The problem with merging three datasets is that crop and weed areas in the target label images are not marked with different colors. For instance, crops in CWFID (Fig. 2.a2) are marked red, but weeds in SB (Fig. 2.b2) and SF (Fig. 2.c2) are marked red as well. In the same manner, weeds in CWFID (Fig. 2.a2) are colored green, whereas crops in SB (Fig. 2.b2) and SF (Fig. 2.c2) are also colored green. Direct use may lead to misclassification of crops and weeds by the model. To correct this, all target label images were normalized with varying colors: carrot (blue), sugar beet (green), sunflower (turquoise), and weeds (red), as depicted in Fig. 2.a3, b3, c3. This is the initial and most crucial step of data pre-processing, which maintains consistency for training.

Another problem with merging the datasets is the imbalanced number of images. CWFID contains 60 images, Sugar Beet has 1800, and Sunflower has 146. If used directly, the model would be biased in favor of the class with the most images. To correct this, CWFID and Sunflower datasets are augmented to be up-sampled, and the Sugar Beet dataset was down-sampled to balance the dataset. This technique, referred to as resampling, provides balanced class distribution. All three data sets were, before re-sampling, divided into 90% training and 10% testing in the hold-out strategy using random sub-sampling^39^ to enhance the model to learn new data.

**Fig. 3:**
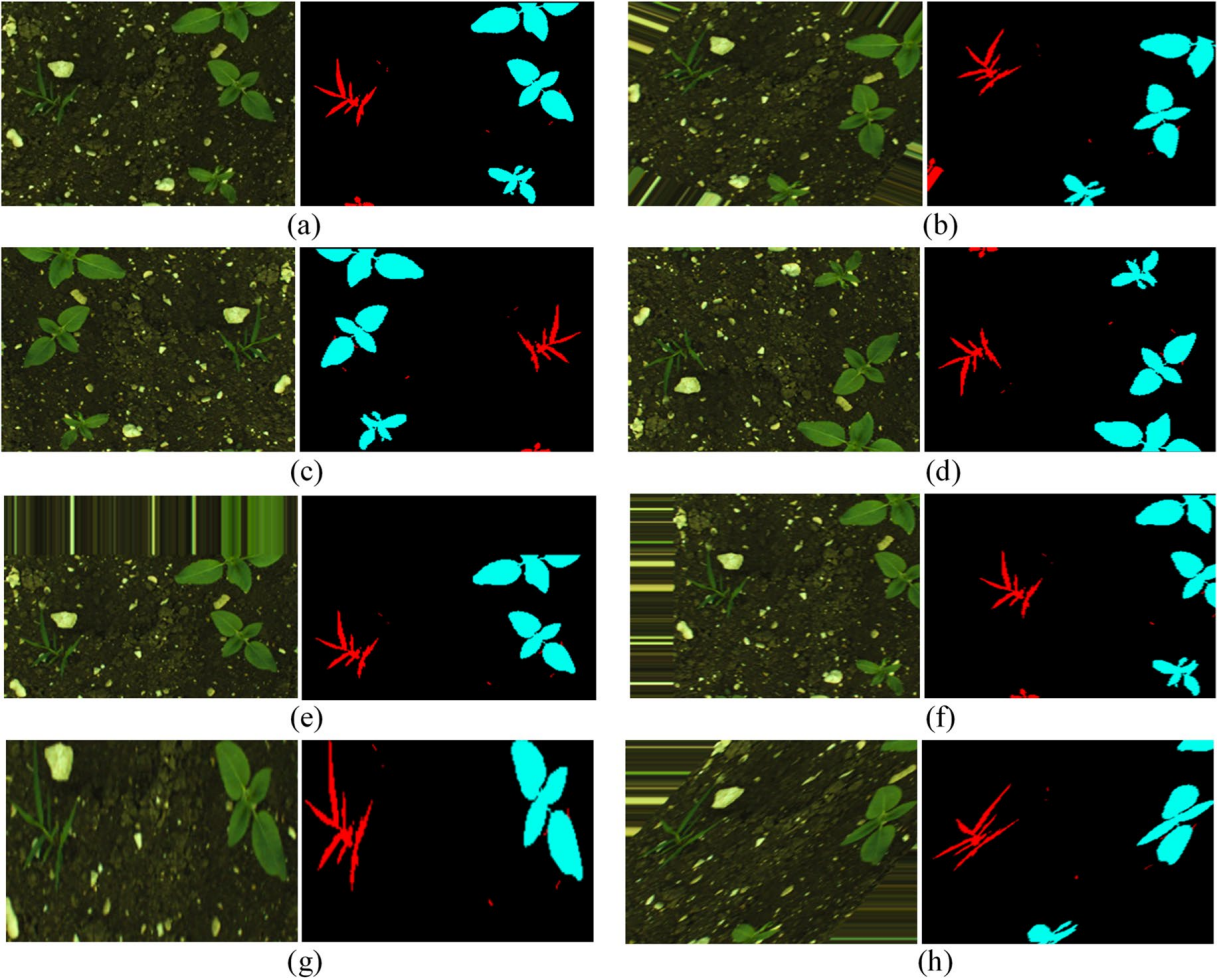
The effect of different augmentation operations on actual field and target label images. (**a**) Original, (**b**) Rotation, (**c**) Horizontal flip, (**d**) Vertical flip, (**e**) Width shift, (**f**) Height shift, (**g**) Zooming, (**h**) Shearing.

During the resampling procedure, random sub-sampling is used to reduce the Sugar Beet dataset to 1100 images, which includes 1000 training images and 100 test images. As mentioned earlier, the augmentation operations are applied to the CWFID and Sunflower datasets. Augmentation techniques such as rotation, zooming, shearing, horizontal and vertical flipping, width and height shifting, and others are applied to both real and target labels in the same order to maintain valid training and testing pairs. Augmentation operations also reduce the likelihood of overfitting by adding variety to the training instances in addition to maintaining an equal distribution of data. As a result of augmentation operations, 54 and 6 images in the training and testing sets of the CWFID dataset were increased to 1000 and 100 images, and 131 and 15 images were increased in the training and testing sets of the Sunflower dataset to 1000 and 100 images, respectively. Hence, with these resampling operations, the number of instances in the three datasets is equalized. Figure 3 shows the effect of these augmentation operations.

The training set is split into an 8:2 proportion using a k-fold cross-validation approach with k = 5 (5-fold cross-validation) to assess the learning progress of each epoch. For each run, four (k-1) of the five parts of the data set are utilized for training, while one is used for validation^40^. Without interfering with the training process, this maintains the model trained on every image. Table 1 lists the characteristics of the multi-crop weed dataset used in this study.

**Table 1:** The details of the multi-crop weed datasets used in this research work.

S. no	Name of the dataset	Actual count	Train and test split		After resampling	
			Train	Test	Train	Test
1	Sugar Beet	1800	1620	180	1000	100
2	CWFID	60	54	6	1000	100
3	Sunflower	146	131	15	1000	100
Total					3000	300

### Proposed concatenated attention UNet with CBAM (CAUC) model

**Fig. 4:**
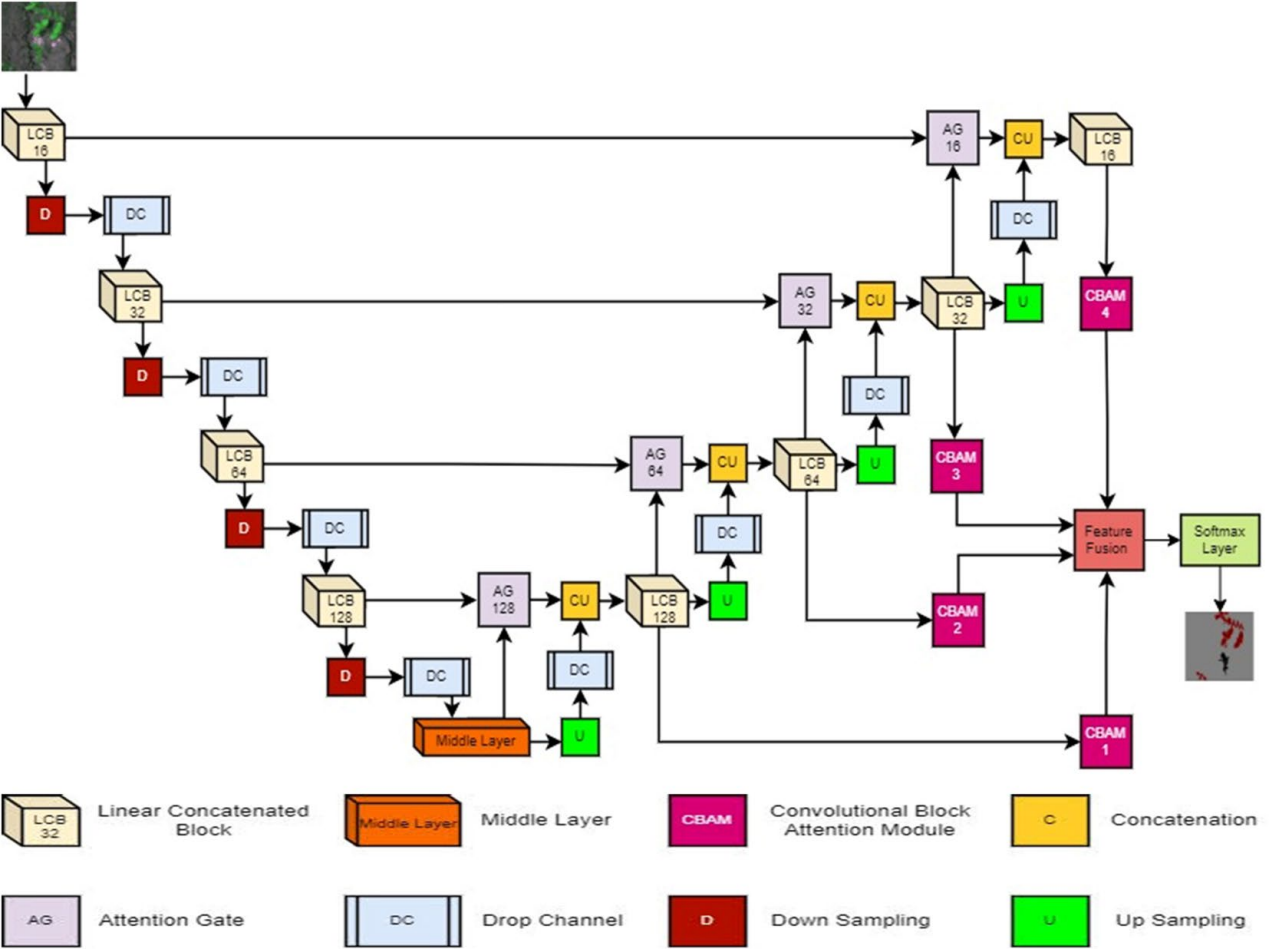
The structure of concatenated attention UNet with CBAM (CAUC).

The proposed Concatenated Attention UNet with CBAM (CAUC) model uses the UNet model as the base structure, an encoder, and a decoder-based model^41^. The proposed model’s structure is depicted in Fig. 4. Linear Concatenated Block (LCB), Convolutional Block Attention Module (CBAM), and Attention Gate (AG) attached skip connections are the key elements of the proposed model and are crucial for extracting the significant features of various crops and weeds. The detailed description and key functionality of the proposed model’s key elements are provided under the following sub-section.

1. Encoder-decoder structure of the proposed CAUC model.
2. Linear concatenated block (LCB).
3. Convolutional block attention module (CBAM).
4. Attention gate (AG) connections.
5. Pixel-wise labelling.

#### Encoder-decoder structure of the proposed CAUC model

The proposed CAUC model incorporates 7 × 7, 3 × 3, and 1 × 1 convolution layers, with 4, 27, and 32 layers, respectively. The 3 × 3 and 1 × 1 convolution layers are predominantly in 8 LCB units and 4 AG components. To alleviate computational complexity, 24 of the 3 × 3 layers in LCB units employ depth-wise separable convolution. The 7 × 7 convolution layers are included in 4 CBAM components. Section 3.2.2 discusses how depth-wise separable convolution alleviates computational complexity.

The two primary blocks of the proposed architectures are the encoder and decoder structures, with a middle block connecting them. The encoder reduces the resolution of important features and extracts them, and the decoder up-samples and restores them to produce the desired segmented image. Both sections have four LCB blocks. The filters of the encoder increase to 12, 32, 64, and 128, and the decoder takes the opposite order. The middle layer includes 256 filters. Unlike RRUDC^10^, an AG-attached skip connection is employed to combine feature maps of encoder and decoder parts, with details in Sect. 4.3.1. To avoid overfitting, a dropout layer is appended after every LCB component, similar to the drop channel method^42^.

Instead of depending on the last LCB component on the decoder side, each LCB component directly contributes to the final output. The CBAM component in the Feature Fusion stage, which collects the most crucial information from the decoder-side LCB feature maps, is used to accumulate the outputs of all LCB components. Section 3.2.4 provides a full description of the CBAM procedure. To precisely segment the crop and weed areas present in the agricultural image, the Softmax layer receives the fused feature maps from the decoder’s LCB components. Table 2 displays the suggested CAUC model’s real design structure.

**Table 2:** The architectural configuration of the proposed CAUC model.

Sec	Input	Component/layer	Kernel size	No of comps /filters	Output and its size	
Encoder SIDE	Image	IP_Conv	3 × 3	64	E1	224 × 224 × 64
	E1	LCB16	1 × 1, 3 × 3	8, 16	E2	224 × 224 × 16
	E2	Down-Samp1	2 × 2	1	E3	112 × 112 × 16
	E3	Drop Chanel	Dropout_rate = 0.2		E4	112 × 112 × 16
	E4	LCB32	1 × 1, 3 × 3	16, 32	E5	112 × 112 × 32
	E5	Down-Samp2	2 × 2	1	E6	56 × 56 × 32
	E6	Drop Chanel	Dropout_rate = 0.2		E7	56 × 56 × 32
	E7	LCB64	1 × 1, 3 × 3	32, 64	E8	56 × 56 × 64
	E8	Down-Samp3	2 × 2	1	E9	28 × 28 × 64
	E9	Drop Chanel	Dropout_rate = 0.5		E10	28 × 28 × 64
	E10	LCB128	1 × 1, 3 × 3	64, 128	E11	28 × 28 × 128
	E11	Down-Samp3	2 × 2	1	E12	14 × 14 × 128
	E12	Drop Chanel	Dropout_rate = 0.5		E13	14 × 14 × 128
	E13	Middle Layer	3 × 3	256	M	14 × 14 × 256
Decoder side	M	Up-Samp1	2 × 2	1	D1	28 × 28 × 256
	D1	Drop Chanel	Dropout_rate = 0.5		D2	28 × 28 × 256
	E11, M	AG128	1 × 1	128	D3	28 × 28 × 128
	D2, D3	ConCat-1	–	1	D3	28 × 28 × 384
	D3	LCB128	1 × 1, 3 × 3	64, 128	D4	28 × 28 × 128
	D4	Up-Samp2	2 × 2	1	D5	56 × 56 × 128
	D5	Drop Chanel	Dropout_rate = 0.5		D6	56 × 56 × 128
	E8, D4	AG64	1 × 1	64	D7	56 × 56 × 64
	D6, D7	ConCat-2	–	1	D8	56 × 56 × 192
	D8	LCB64	1 × 1, 3 × 3	32, 64	D9	56 × 56 × 64
	D9	Up-Samp3	2 × 2	1	D10	112 × 112 × 64
	D10	Drop Chanel	Dropout_rate = 0.2		D11	112 × 112 × 64
	E5, D9	AG32	1 × 1	32	D12	112 × 112 × 32
	D11,D12	ConCat-3	–	1	D13	112 × 112 × 96
	D13	LCB32	1 × 1, 3 × 3	16, 32	D14	112 × 112 × 32
	D14	Up-Samp4	2 × 2	1	D15	224 × 224 × 32
	D15	Drop Chanel	Dropout_rate = 0.2		D16	224 × 224 × 32
	E2, D14	AG16	1 × 1	32	D17	224 × 224 × 16
	D16,D17	ConCat-4	–	1	D18	224 × 224 × 48
	D18	LCB16	1 × 1, 3 × 3	8. 16	D19	224 × 224 × 16
Feature fusion	D4	CBAM1 + UpSmap	7 × 7, 8 × 8	1	F1	224 × 224 × 16
	D9	CBAM2+ UpSmap	7 × 7, 4 × 4	1	F2	224 × 224 × 64
	D14	CBAM3+ UpSmap	7 × 7, 2 × 2	1	F3	224 × 224 × 32
	D19	CBAM6	7 × 7	1	F4	224 × 224 × 16
	F1, F2, F3, F4	ConCat-5	–	1	F5	224 × 224 × 240
	F5	OP_Conv	3 × 3	5	F6	224 × 224 × 5
	F6	Softmax Layer	–	–	F7	224 × 224 × 5

#### Linear concatenated block (LCB)

**Fig. 5:**
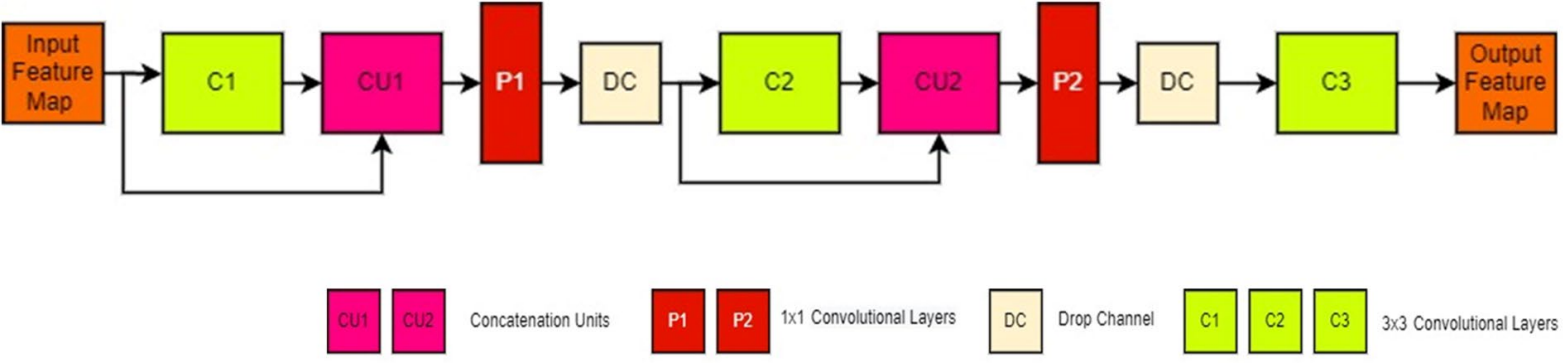
The structure of linear concatenated block (LCB).

The Linear Concatenated Block (LCB) is an crucial component of the CAUC model. It comprises two 1 × 1 and three 3 × 3 convolutional layers, with subsequent ReLU activation and Batch Normalization (BN). The LCB architecture facilitates the efficient utilization of feature maps at minimal computational expense. This is done through linear concatenation of convolution layers and application of 1 × 1 convolutions combined with depth-wise separable convolution to minimize complexity. The LCB’s internal structural components are depicted in Fig. 5, and its mathematical model in Eqs. 1–7 and LCB64 configuration in Table 3.

Linear concatenation is an efficient computation method for employing feature maps in a CNN model as opposed to global concatenation. Every LCB level consists of two concatenation units (CU1, CU2) (Fig. 5), which enable the C2 and C3 convolution layers to take in both the input and output of the preceding component (Eqs. 4 and 7). This helps in segmenting the crop and weed portions in the agricultural field images. Yet, concatenation adds computational parameters by increasing the channel size (Eqs. 2 and 5).

1 $${{\mathrm{C}}_{\mathrm{1}}}\,=\,{{\mathrm{W}}_{\mathrm{1}}} \otimes {\mathrm{X}}({\text{Size of C1}}:{\text{ H}} \times {\mathrm{W}} \times {\mathrm{C}}{{\mathrm{h}}_{\mathrm{1}}})$$

2 $${\mathrm{C}}{{\mathrm{U}}_{\mathrm{1}}}\,=\,{\text{ConCat }}({\mathrm{C}},{{\mathrm{C}}_{\mathrm{1}}})\,({\text{Size of CU1}}:{\text{ H}} \times {\mathrm{W}} \times ({\mathrm{Ch}}\,+\,{\mathrm{C}}{{\mathrm{h}}_{\mathrm{1}}}))$$

3 $${\mathrm{P1}}={{\mathrm{W}}_{\mathrm{a}}} \odot {\mathrm{C}}{{\mathrm{U}}_{\mathrm{1}}}\,\,({\text{Size of P1}}:{\text{ H}} \times {\mathrm{W}} \times {\mathrm{N}})$$

4 $$\begin{gathered} {{\mathrm{C}}_{\mathrm{2}}}={{\mathrm{W}}_{\mathrm{2}}}~ \otimes {\mathrm{P1}}\,({\text{Size of C2}}:{\text{ H}} \times {\mathrm{W}} \times {\mathrm{C}}{{\mathrm{h}}_{\mathrm{2}}}) \hfill \\ ={{\mathrm{W}}_{\mathrm{2}}}~ \otimes ({{\mathrm{W}}_{\mathrm{a}}} \odot {\mathrm{CU1}}) \hfill \\ ={{\mathrm{W}}_{\mathrm{2}}}~ \otimes ({{\mathrm{W}}_{\mathrm{a}}} \odot {\text{ConCat }}({\mathrm{C}},{{\mathrm{C}}_{\mathrm{1}}})) \hfill \\ \end{gathered}$$

5 $${\mathrm{C}}{{\mathrm{U}}_{\mathrm{2}}}\,=\,{\text{ConCat }}({\mathrm{P1}},{{\mathrm{C}}_{\mathrm{2}}}){\text{ }}(Size{\text{ }}of{\text{ }}CU2:{\text{ }}H \times W \times (N\,+\,Ch2)$$

6 $${\mathrm{P2}}\,=\,{{\mathrm{W}}_{\mathrm{b}}} \odot {\mathrm{C}}{{\mathrm{U}}_{\mathrm{2}}}({\text{Size of P2 }}:{\text{ H}} \times {\mathrm{W}} \times {\mathrm{N}})$$

7 $$\begin{gathered} {{\mathrm{C}}_{\mathrm{3}}}\,=\,{{\mathrm{W}}_{\mathrm{3}}}~ \otimes {\mathrm{P2}}(Size{\text{ }}of{\text{ }}C3{\text{ }}H \times W \times C{h_3}) \hfill \\ ={{\mathrm{W}}_{\mathrm{3}}}~ \otimes ({{\mathrm{W}}_{\mathrm{b}}} \odot {\mathrm{C}}{{\mathrm{U}}_{\mathrm{2}}}) \hfill \\ ={{\mathrm{W}}_{\mathrm{3}}} \otimes ~({{\mathrm{W}}_{\mathrm{b}}} \odot {\text{ConCat }}({\mathrm{P1}},{{\mathrm{C}}_{\mathrm{2}}})) \hfill \\ \end{gathered}$$

where, C_1_, C_2_, C_3_, - the convolution layers’ outcome, PC_a_, PC_b_ - the 1 × 1 convolution layers’ outcome, W_1_, W_2_, W_3_ – Weights of the convolution layers, W_a_, W_b_, – Weights of the 1 × 1 convolution layers, CU_1_, CU_2_ - the Concatenation layers’ outcome, H, W – Feature map Height and Width, Ch_1_, Ch_2_, Ch_3_ – Channel size of the generated feature maps, C – input feature map for the respective LCB, Ch - the respective input feature map’s Channel size of the LCB component, ʘ − 1 × 1 convolution operator, $\:\otimes\:$ - convolution operator, ConCat() - Concatenation operations.

**Table 3:** The architectural configuration of LCB64.

Section	I/*P*	Component/ layer	Kernel size	No of components/filters	Output size	
LCB64	IF = 56 × 56 × 32	Tres_DSC1	3 × 3	64	L1	56 × 56 × 64
	IF, L1	ConCat1	–	1	L2	56 × 56 × 96
	L2	Unus_SC1	1 × 1	32	L3	56 × 56 × 32
	L3	Drop_Layer	Dropout_rate = 0.1		L4	56 × 56 × 32
	L4	Tres_DSC2	3 × 3	64	L5	56 × 56 × 64
	L3, L5	ConCat1	–	1	L6	56 × 56 × 96
	L6	Unus_SC2	1 × 1	32	L7	56 × 56 × 32
	L7	Drop_Layer	dropout_rate = 0.1		L8	56 × 56 × 32
	L8	Tres_DSC3	3 × 3	64	L9	56 × 56 × 64

Tres_DSC : 3 × 3 DSC layer, Unus_SC : 1 × 1 Standard Convolution layer.

The overall number of computational parameters present in the model decides its computational complexity. Consequently, as the model’s computational parameters are reduced, its computational complexity and size fall automatically. This is accomplished in two ways in this research work: Parameter Reduction through depth-wise separable convolution layers - PR1 and Parameter Reduction through 1 × 1 convolution layers - PR2.

The fundamental operation of CNN is the convolution operation. Larger filter sizes in convolution aid in extracting discriminative features from intricate objects, enhancing performance. But the drawback of regular convolution is the increased computational cost with filters of size K×K×IC, where K is the Kernel Size and IC is the Number of Channels in the feature map. A more effective method of decreasing computational parameters without impacting performance is depth-wise separable convolution (DSC) operations. Thus, the middle layer and all 3 × 3 convolution layers in LCB components are designed as depth-wise separable convolution layers, which constitute the first parameter reduction strategy.

The DSC operation can be achieved in two steps to reduce the computational parameters. In the initial step, features at the spatial level are obtained through depth-wise convolution operations, and in the second step, features at the channel level are obtained through point-wise convolution operations^43^. Through this strategy, the expensive convolution filter K×K×IC is decomposed into two components, K×K×1 for the first step and 1 × 1×IC for the second step of the DSC operation. The mathematical notation for the number of computational parameters produced by the regular convolution and DSC computations, as well as parameter decrease per layer employing DSC layers, is illustrated in Eqs. 8–11.

8 $${\mathrm{N}}{{\mathrm{P}}_{{\mathrm{SC}}}}={\mathrm{K}} \times {\mathrm{K}} \times {\mathrm{IC}} \times {\mathrm{NF}}$$

9 $${\mathrm{N}}{{\mathrm{P}}_{{\mathrm{DSC}}}}=({\mathrm{K}} \times {\mathrm{K}} \times {\mathrm{IC}})+({\mathrm{IC}} \times {\mathrm{N}})$$

$$\:{\mathbf{P}\mathbf{R}}_{1}\:=\frac{\begin{array}{c}Total\:number\:of\:computational\:parameters\:produced\:by\:\\\:a\:depth-wise\:separable\:convolution\:layer\left({\mathrm{N}\mathrm{P}}_{\mathrm{D}\mathrm{S}\mathrm{C}}\right)\end{array}}{\begin{array}{c}Total\:number\:of\:computational\:parameters\:produced\:by\:\\\:a\:standard\:convolution\:layer\:\left({\mathrm{N}\mathrm{P}}_{\mathrm{S}\mathrm{C}}\right)\end{array}}$$

$$\:=\frac{({\mathrm{K}}^{2}\times\:\mathrm{I}\mathrm{C})\:+\:(\mathrm{I}\mathrm{C}\times\:\:\mathrm{N})}{{\mathrm{K}}^{2}\times\:\mathrm{I}\mathrm{C}\times\:\:\mathrm{N}}$$

$$\:=\frac{\mathrm{I}\mathrm{C}({\mathrm{K}}^{2}+\mathrm{N})}{\:{\mathrm{K}}^{2}\times\:\mathrm{I}\mathrm{C}\times\:\mathrm{N}}$$

$$\:=\frac{1}{{\mathrm{K}}^{2}}+\frac{1}{\mathrm{N}}(Since,\:K\:\hspace{0.17em}=\:\hspace{0.17em}3)$$

10 $$\:{\mathrm{P}\mathrm{R}}_{1}=\frac{1}{9}+\frac{1}{\mathrm{N}}$$

11 $$\:{\mathrm{A}\mathrm{P}\mathrm{R}}_{1}=\frac{\sum\:_{i=1}^{m}{PR}_{1i}}{m}$$

where PR_1_ – Parameter Reduction rate per layer using the DSC operation, APR_1_ – Average Parameter Reduction rate using the DSC layer, K – Kernel size, IC – Input feature map’s Channels size, and N – the convolution layers’ filters size, m – Number of convolution layers used in the LCB components and middle layer. The lowest and highest numbers of filters used in CAUC are 16 and 256, respectively. Hence, the maximum and minimum parameter reduction per layer using the DSC layer (PR_1_) can be achieved in the range of 12% to 17% on *N* = 256 and *N* = 16, respectively. The Average Parameter Reduction using DSC operation (APR_1_) of ~ 14% is achieved on the proposed CAUC model with these DSC layers.

The computational parameters are linearly proportional to the input feature map’s channels. Feature maps are used efficiently by the concatenation unit, but they raise channel size, resulting in increased computational complexity (given in Eqs. 2 and 5). For this, a 1 × 1 convolution layer is introduced after the concatenation unit for cross-channel down-sampling, making the second parameter reduction (PR2) strategy in this work. Consequently, the output of the concatenation unit is passed through the 1 × 1 layer to restrict channel size (in Eqs. 3 and 6). For further computational complexity reductions, the 1 × 1 convolution layer’s number of filters is fixed at fewer than the input feature map’s channels. Hence, the filters in the 1 × 1 convolution are maintained at half (N/2) of the allocated filters per LCB unit. The parameter reduction rate for each LCB component (PR2) is computed mathematically in Eq. 12.

$$\:\:\:\:\:\:\:\:\:\:\:\:{\mathbf{P}\mathbf{R}}_{2}=\frac{\begin{array}{c}Total\:number\:of\:compuational\:\:parameters\:produced\:in\:a\:LCB\:unit\:\\\:that\:employs\:1\times\:1\:\:convolution\:layer\end{array}}{\begin{array}{c}Total\:number\:of\:computational\:parameters\:produced\:in\:a\:LCB\:unit\:\\\:that\:does\:not\:employ\:1\times\:1\:convolution\:layer\end{array}}$$

$$\:=\frac{\mathrm{N}\left(\mathrm{C}1\right)+\mathrm{N}\left(\mathrm{P}1\right)+\mathrm{N}\left(\mathrm{C}2\right)+\mathrm{N}\left(\mathrm{P}2\right)+\mathrm{N}\left(\mathrm{C}3\right)}{\mathrm{N}\left(\mathrm{C}1\right)+\mathrm{N}\left(\mathrm{C}2\right)+\mathrm{N}\left(\mathrm{C}3\right)}$$

$$\:=\frac{\left[\left[{(K}^{2}\times\:Ni\right)+\left(NNi\right)\right]+\left[(N+Ni)\times\:\frac{N}{2}\right]+\left[\left({(K}^{2}\times\:\frac{N}{2}\right)+(\frac{N}{2}\times\:N)\right]+\left[\frac{3N}{2}\times\:\frac{N}{2}\right]+[\left({(K}^{2}\times\:\frac{N}{2}\right)+(\frac{N}{2}\times\:N\left)\right]}{\left[\left[{(K}^{2}\times\:Ni\right)+\left(NNi\right)\right]+\left[{(K}^{2}\times\:(N+Ni\right))+(N+Ni\left)N\right]+\left[\left[{(K}^{2}\times\:(2N+Ni\right))+(N+Ni)N\right]}$$

$$\:=\frac{\mathrm{N}\mathrm{i}{\times\:\mathrm{K}}^{2}+\left(\mathrm{N}\mathrm{N}\mathrm{i}\right)]+\frac{\mathrm{N}(\mathrm{N}+\mathrm{N}\mathrm{i})}{2}+\frac{\mathrm{N}{\mathrm{K}}^{2}}{2}+\frac{{\mathrm{N}}^{2}}{2}+\frac{3{\mathrm{N}}^{2}}{2}+\frac{\mathrm{N}{\mathrm{K}}^{2}}{2}+\frac{{\mathrm{N}}^{2}}{2}}{\mathrm{N}{\mathrm{K}}^{2}+\mathrm{N}\mathrm{N}\mathrm{i}+{\mathrm{K}}^{2}\left(\mathrm{N}+\mathrm{N}\mathrm{i}\right)+\mathrm{N}\left(\mathrm{N}+\mathrm{N}\mathrm{i}\right)+{\mathrm{K}}^{2}\left(2\mathrm{N}+\mathrm{N}\mathrm{i}\right)+\mathrm{N}\left(2\mathrm{N}+\mathrm{N}\mathrm{i}\right)}$$

$$\:=\frac{{\mathrm{K}}^{2}\left(\mathrm{N}+\mathrm{N}\mathrm{i}\right)+\frac{3}{4}{\mathrm{N}}^{2}+\frac{3}{2}{\mathrm{N}}^{2}+\frac{3}{2}\mathrm{N}\mathrm{N}\mathrm{i}}{3{\mathrm{K}}^{2}\left(\mathrm{N}+\mathrm{N}\mathrm{i}\right)+3\mathrm{N}(\mathrm{N}+\mathrm{N}\mathrm{i})}$$

$$\:=\frac{{\mathrm{K}}^{2}\left(\mathrm{N}+\mathrm{N}\mathrm{i}\right)+\frac{3\mathrm{N}}{2}\left(\mathrm{N}+\mathrm{N}\mathrm{i}\right)+\frac{3}{4}{\mathrm{N}}^{2}}{3\left(\mathrm{N}+\mathrm{N}\mathrm{i}\right)\left({\mathrm{K}}^{2}+\mathrm{N}\right)}$$

$$\:=\frac{{\mathrm{K}}^{2}+3\mathrm{N}}{6({\mathrm{K}}^{2}+\mathrm{N})}+\:\frac{{\mathrm{N}}^{2}}{4(N+Ni)({\mathrm{K}}^{2}+N)}$$

12 $$\:{\mathbf{P}\mathbf{R}}_{2}=\frac{1}{\left(9+N\right)}(3+\frac{N}{2}+\frac{{\mathrm{N}}^{2}}{4\left(\mathrm{N}+\mathrm{N}\mathrm{i}\right)})$$

where PR_2_ – Parameter Reduction Rate per LCB component using the 1 × 1 convolution layer, K – Kernel size, IC - Input feature map’s Channels size, N – the convolution layers’ filters size, Ni – Input channel size of the initial input feature map for an LCB component. The Ni is derived from the number of filters employed in the earlier LCB part of the model. The filter allocation of the LCB part is 16, 32, 64, and 128 on the encoder side, and the same pattern is mirrored on the decoder side. We therefore assume that Ni and N are X and 2X on the encoder side and 2X and X on the decoder side. The PR2 equation described in Eq. 12 is again given as PR2E (encoder side) and PR2D (decoder side) as described in Eqs. 13 and 14. The mathematical formula for the Average Parameter Reduction rate (APR2) per LCB component through the 1 × 1 convolution layer is given in Eq. 15.

13 $$\begin{aligned} & {\mathbf{P}\mathbf{R}}_{2\mathbf{D}}\:=\frac{1}{\left(9+2x\right)}+\left(3+x+\frac{4{x}^{2}}{12x}\right)\:(where\:{N}_{i}=x,\:N=2x\nonumber\\ &= \frac{1}{3}+\frac{2x}{3(9+2x)} \end{aligned}$$

14 $$\begin{aligned} & {\mathbf{P}\mathbf{R}}_{2\mathbf{D}}\:=\frac{1}{\left(9+x\right)}+\left(3+\frac{x}{2}+\frac{{x}^{2}}{12x}\right)\:(where\:{N}_{i}=2x,\:N=x)\nonumber\\ &=\frac{1}{3}+\frac{x}{4(9+x)}\end{aligned}$$

15 $$\:{\mathbf{A}\mathbf{P}\mathbf{R}}_{2\mathbf{D}}=\frac{\sum\:_{i=1}^{NE}{PR}_{2Ei}\:+\:\sum\:_{j=1}^{ND}{PR}_{2Dj}}{\mathrm{N}\mathrm{E}+\mathrm{N}\mathrm{D}}$$

where, APR_2_ – Average Parameter Reduction rate using per LCB component using the 1 × 1 convolution layer, and NE, ND – Number of LCB components present in the encoder and decoder side, respectively. The APR_2_ of ~ 58% is achieved in the proposed CAUC model using the 1 × 1 convolution layer, where APR_2E_ and APR_2D_ are ~ 63.5% and ~ 52.5%, respectively. To restrain the model from overfitting, a dropout layer is added after the 1 × 1 convolution layer as a drop-layer mechanism^42^.

The LCB component is playing a significant role in computational complexity reductions by preserving performance through the use of lightweight depthwise separable convolution operations instead of standard convolution operations (APR_1_ = ~ 14%). Further, the design choice includes the integration of a 1 × 1 convolution layer after the concatenation operations, streamlining feature integration and minimizing computational overhead (APR_2_ of = ~ 58%).

#### Attention gate (AG) attached skip connections

**Fig. 6:**
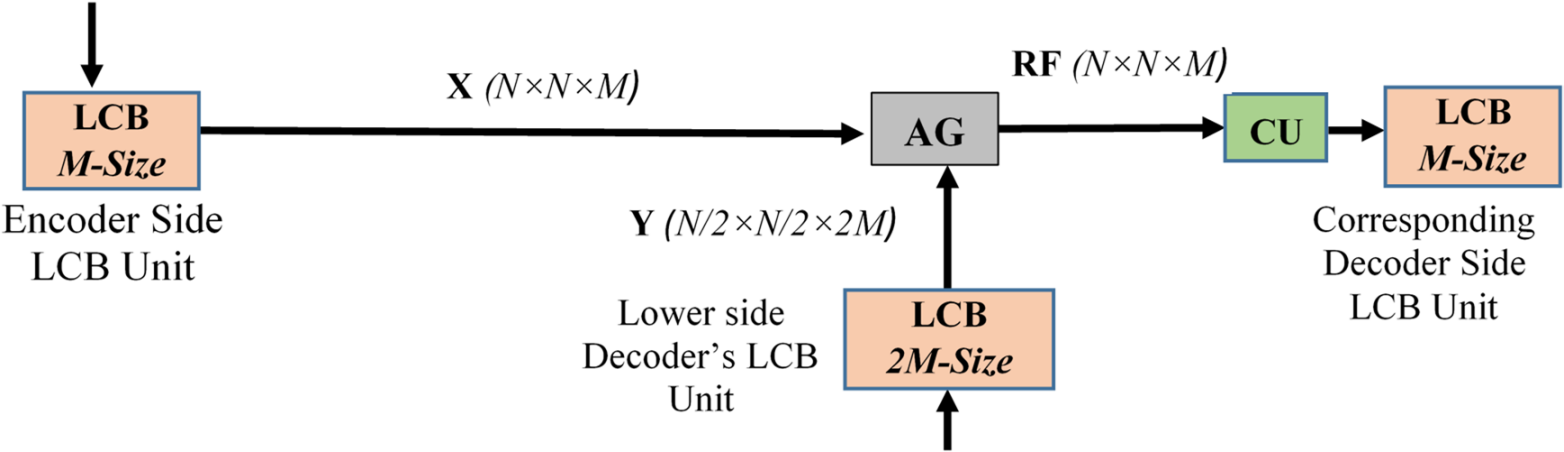
The structure of the AG attached skip connection that connects the encoder and decoder blocks.

The standard UNet typically integrates feature maps produced by encoder blocks with the corresponding decoder blocks via the skip connection, which adds minimal redundant spatial information from the encoder side. In view of overcoming this issue, an Attention Gate (AG) attached a skip connection is used in this proposed CAUC model. Soft attention is used in this case to minimize redundant features and suppress the activations of irrelevant regions. Furthermore, the AG-attached skip connection in the proposed CAUC model integrates the feature maps generated by the encoder-side LCB Unit and the corresponding lower-side decoder’s LCB unit with the respective decoder-side LCB unit. It helps to enhance the representation ability of the model. The structure of the AG attached skip connection is shown in Fig. 6.

**Fig. 7:**
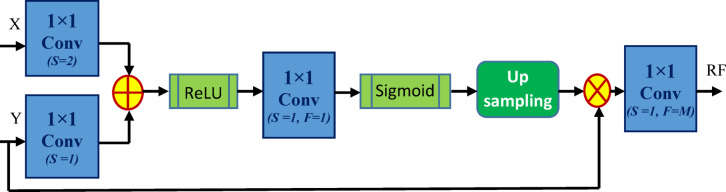
The structure of the Attention Gate(AG) used in the AG-attached skip connection of the proposed CAUC model. The size of X, Y, and RF are (N×N×M), ( $\:\frac{N}{2}$×$\:\frac{N}{2}$×$\:2M$) and (N×N×M), respectively.

**Figure:**
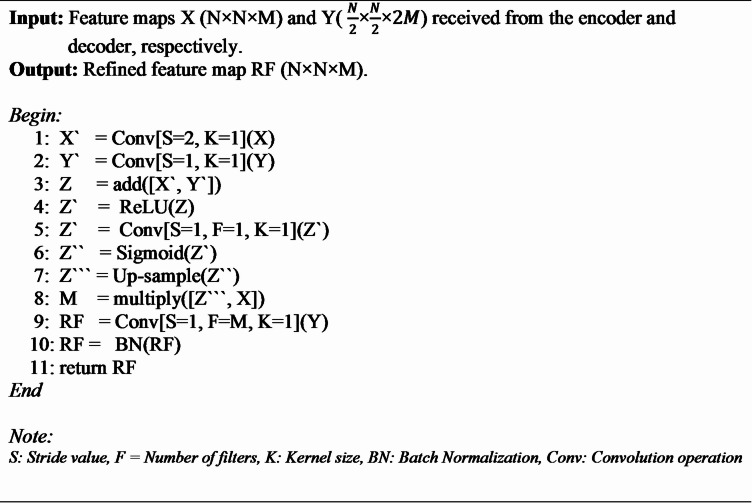
**Algorithm 1** Attention Gate.

There are two inputs, X and Y, for an attention gate (AG), in which Y is received from the deeper part of the model and its smaller size ($\:\frac{N}{2}$×$\:\frac{N}{2}$×$\:2M$) when compared to X (N×N×M). The detailed functionality of AG is mentioned in Algorithm 1 and also represented in Fig. 7. The significant activations in the input feature maps are highlighted after the various stages of refinement described in Algorithm 1, and the refined feature map is then provided to the appropriate decoder blocks through the concatenation unit. It assists the proposed CAUC model in focusing on significant features specific to the task^44,45^.

Overall, the AG-attached nested skip connections present in the proposed CAUC model significantly help to attain better performance on multi-crop segmentation by transferring the distinct crops (Carrot, SugarBeet, and Sunflower) and weed–specific features and filtering out irrelevant background details when compared to conventional skip connections.

#### Convolutional block attention module (CBAM)

CBAM is one of the lightweight and effective attention modules that can be attached to any CNN model without significantly increasing its computational complexity. The key role of the CBAM module is to interpret the input feature map spatially and channel-wise and finally multiply these interpretations to produce the most distinguishable refined feature map^46^. It makes the proposed model focus on significant features.

**Fig. 8:**
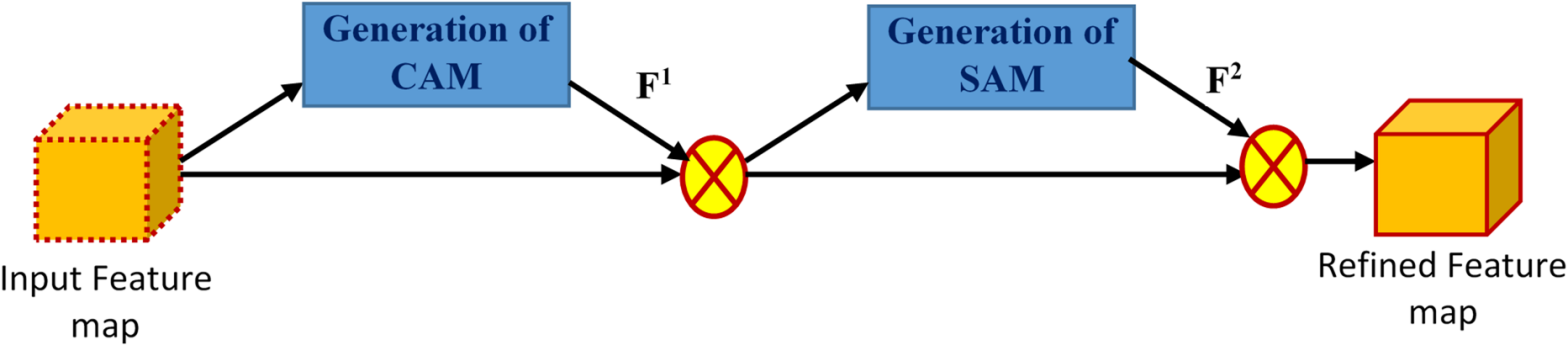
The structure of the convolutional block attention module (CBAM) used in the proposed CAUC model.

The two attention modules, i.e., the Channel Attention Module (CAM) and the Spatial Attention Module (SAM), are put sequentially^23^. The organization of CBAM and its sequential arrangement of the attention modules are depicted in Fig. 8. The mechanism of CBAM is explained in Algorithm 2 and mathematically formulated in Eqs. 16–18.

$$\begin{gathered} {\mathrm{OP}}\left( {{\mathrm{CBAM}}} \right)\,=\,{\mathrm{SAM}}\left( {{\mathrm{CAM}}\left( {\mathrm{F}} \right)} \right) \hfill \\ {\mathrm{CAM}}\left( {\mathrm{F}} \right)\,=\,{{\mathrm{F}}^{\mathrm{1}}} \hfill \\ ={{\mathrm{M}}_{{\mathrm{CH}}}}\left( {\mathrm{F}} \right) \odot {\mathrm{F}}\,=\,{{F^{\prime}}} \hfill \\ \end{gathered}$$

$$\begin{gathered} {\mathrm{SAM}}\left( {{\mathrm{CAM}}\left( {\mathrm{F}} \right)} \right)\,=\,{{\mathrm{F}}^{\mathrm{2}}} \hfill \\ ={\text{ SAM}}\left( {{{\mathrm{F}}^{\mathrm{1}}}} \right)={{\mathrm{M}}_{{\mathrm{SP}}}}\left( {{{\mathrm{F}}^{\mathrm{1}}}} \right) \odot {{\mathrm{F}}^{\mathrm{1}}} \hfill \\ \end{gathered}$$

16 $${\mathrm{OP}}\left( {{\mathrm{CBAM}}} \right)={{\mathrm{M}}_{{\mathrm{SP}}}}\left( {{{\mathrm{F}}^{\mathrm{1}}}} \right) \odot {{\mathrm{M}}_{{\mathrm{CH}}}}\left( {\mathrm{F}} \right) \odot {\mathrm{F}}$$

17 $${{\mathrm{M}}_{{\mathrm{CH}}}}\left( {\mathrm{F}} \right)=\sigma ({\text{MLP }}\left( {{{\mathrm{F}}^{{\mathrm{ch}}}}_{{{\mathrm{avg}}}}} \right)+{\text{MLP }}\left( {{{\mathrm{F}}^{{\mathrm{ch}}}}_{{{\mathrm{max}}}}} \right)$$

18 $${{\mathrm{M}}_{{\mathrm{SP}}}}\left( {{{\mathrm{F}}^{\mathrm{1}}}} \right)=\sigma ({\mathrm{7}} \times {\mathrm{7}} - {\text{Conv }}\left( {{{\mathrm{F}}^{{\mathrm{sp}}}}_{{{\mathrm{avg}}}};{\text{ }}{{\mathrm{F}}^{{\mathrm{sp}}}}_{{{\mathrm{max}}}}} \right)$$

where, O – Output of a CBAM, F – dimension of input Feature map (H×W×C), M_CH_ – dimension of channel attention map (1 × 1×C), M_SP_ – dimension of spatial attention map (H×W×1), ʘ - Element-wise multiplication, MLP – Shared multi-layer perceptron with one hidden layer, F^ch^_avg_, F^ch^_max_ - Average and max pooling of channel attention map, F^sp^_avg_, F^sp^_max_ - Average and max pooling of spatial attention map, σ – Sigmoid function, 7 × 7-Conv – Convolution operation of kernel size 7.

As visualized in Fig. 4, employing CBAM after each of the decoder blocks (LCB16, LCB32, LCB64, and LCB128) facilitates the proposed CAUC to process the best discriminative spatial and channel features at various levels of decoding rather than using the output of the final decoder block alone (LCB16) like the standard UNet. By fusing the CBAM-refined feature maps from every decoder block, the framework can take advantage of rich multi-scale contextual information in such a way that fine-grained crop–weed boundaries and large structural patterns are preserved. This design choice helps to generate a more accurate and robust multi-crops and weed segmentation than generating the segmented image only from the final decoder block.

**Figure:**
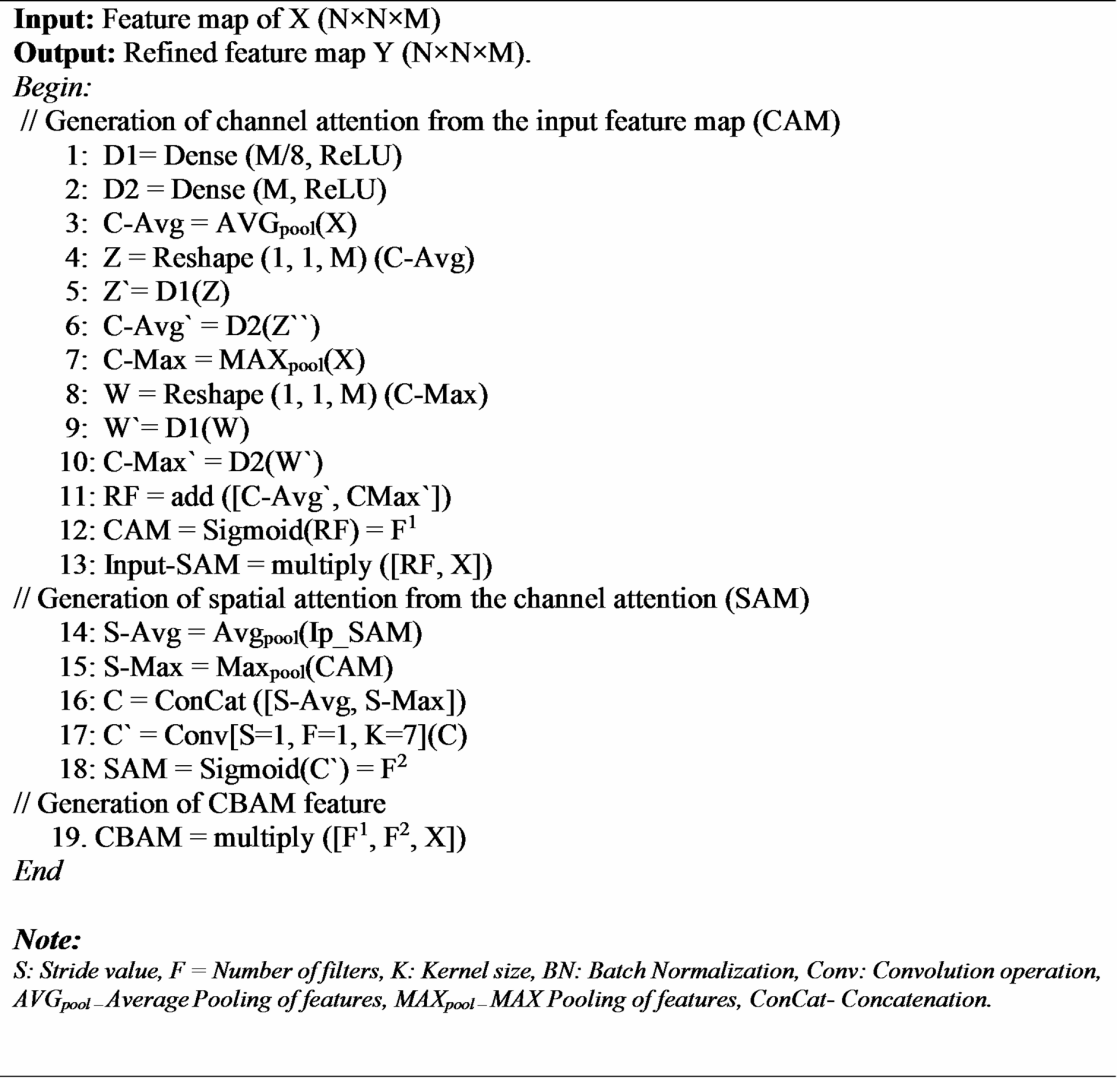
**Algorithm 2** CBAM.

#### Pixel-wise labelling

A technique for examining and categorizing each pixel in an image is called “pixel-wise labeling,” in which each pixel is given a unique interpretation and class^24,47^. Effective crop and weed segmentation requires the use of this approach. Five types of objects may be found in the images utilized in this study: background soil, weeds, sunflower crops, sugar beet crops, and carrot crops. Thus, {carrot, sugar beet, sunflower, weed, soil} = {0, 1, 2, 3, 4} are the class values for these objects, and the model predictions fall into one of these classes. This technique is made easier by converting the label images into a one-hot encoding format, which allows field images to be precisely segmented into crop and weed sections.

### Building a computer vision application

Using the proposed CAUC model as an underlying core component, a computer vision application for a multi-crop and weed segmentation model is developed. The proposed CAUC model has been designed in such a way as to produce a better performance at a lesser computational complexity, where the final model size and the number of computational parameters generated by the model are ~ 5.6 MB and 0.37 million, respectively. The design of this computer vision app utilizes the resources of mobile devices to run the computer vision application. This design method helps to assess how fast the proposed model works on resource-restricted edge/devices.

**Fig. 9:**
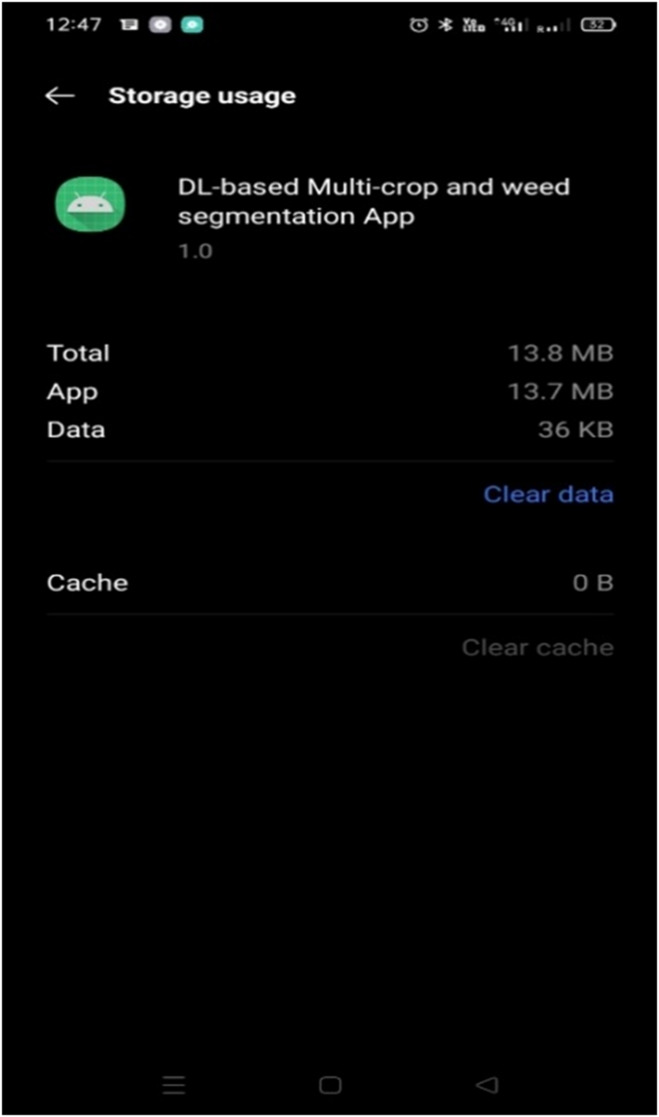
The memory consumption of the developed DL-based multi-crop and weed segmentation App on a mobile device.

TensorFlow Lite is one of the cross-platform frameworks for building deep learning-based computer vision applications to deploy on resource-restricted devices like mobile phones and other embedded devices^48^. In addition to TensorFlow Lite conversion, with the quantization techniques, the model size gets reduced further without affecting its performance. Finally, the reduced size is 1.5 MB. With this CAUC model in TensorFlow Lite format, a DL-based multi-crop weed segmentation App of 13.7 MB is developed. Its memory consumption on the mobile device is shown in Fig. 9.

Brendon Boshell states that the average mobile app published on the app stores is between 11.5 MB and 14.6 MB^49^. The size of the proposed DL-based computer mobile application lies within this range, that is, 13.7 MB.

## Design of experimental settings and evaluation strategies

### Experimental settings

The proposed CAUC model and the other state-of-the-art CNN models utilized in this study are implemented using the Keras and TensorFlow frameworks on the Google Colab platform. Google Colab is an online Jupyter Notebook that runs the code on the cloud through the browser. It offers CUDA Version 11.2, NVIDIA Tesla T4 GPU, 13 GB of RAM, and 68 GB of virtual disc space. It can, therefore, be operated on any computer with an internet connection and is not hardware-restricted.

The dataset used to build and train the proposed CAUC and other state-of-the-art CNN models is created by integrating the three datasets, namely CWFID^37^, Sugar Beet^38^, and Sunflower^27^. Initially, the dataset is split into a 9:1 ratio for building training and testing datasets using the holdout strategy before the resampling process. Following the split of the train and test datasets, the resampling process brings all three datasets to the desired and equal numbers. During this resampling process, the augmentation process is applied to the CWFID and Sunflower datasets, and random sub-sampling is applied to the Sugar Beet dataset, to increase and decrease the instances of the dataset, respectively. Later, to assess the performance in every training epoch, the training dataset is further divided into train and validation datasets using the cross-validation strategy with a validation split of 0.2. A pair of actual crop field images and their corresponding target label images present in the training dataset is used to train the proposed CAUC model, and all these images are resized to the size of 224 × 224 × 3. The proposed CAUC and other deep learning models implemented in this work for performance evaluation, such as SegNet512, UNet, RRUDC, UNet++, and DeepLabV3+, are trained for 50 epochs, where batch_size is 8.

Drop-layer and drop-channel are two algorithms used to add dropout layers to avoid overfitting^42^. A 10% dropout rate is implemented within each LCB component in the drop-layer method. In the drop-channel approach, the top and bottom levels of the LCB components in the proposed CAUC model are followed by dropout rates of 10% and 20%, respectively.

The cross-entropy loss between the pixel values present in the target label and the generated segmented image is measured using the categorical cross-entropy loss function. To do so, all the label images are converted into one-hot encoding form. To optimize these deviations between the target label and the generated segmented image, the ADAM optimizer with a learning rate of 0.001 is used. Using the TensorFlow Lite converter, the trained CAUC model is converted into TensorFlow Lite format. Later, a computer vision application is developed from this converted CAUC model using the Android Studio IDE.

### Evaluation metrics

In terms of the model’s efficiency comparison, we evaluate its performance in segmenting crop and weed portions and its suitability for resource-restricted devices using the following eight metrics. There are.

1. Accuracy (A).
2. Loss (L).
3. Precision (P).
4. Recall (R).
5. F1 score (FS).
6. Mean IoU (MIoU).
7. Mean response time (MRT).
8. Number of parameters generated by the model (NPM).
9. Model size (S).

Accuracy(A) states that the ratio of correctly classified pixels in the crop and weed segmented images is identified correctly to the total number of pixels in the crop and weed segmented images. Using the cross-entropy loss function, the loss (L) is determined by the difference between the actual target pixel value and the pixel value predicted by the model. The precision (P) is expressed as the fraction of correctly identified images’ pixels to the actual images’ positive pixels. The ratio between the actual positive predicted pixels and the identified pixels correctly is expressed as Recall (R). F1-score (FS) is a metric that combines precision and recall produced by the model, also called the harmonic mean of both values. The MIoU is the mean of IoU’s overall area of interest. An IoU measure assesses how well the generated crop and weed-segmented images correspond to the target labels of the crop and weed portions. The Mean Response Time (MRT) has been referred to as the average of the response time taken for segmenting the crop and weed pixels in the actual crop field images. The overall number of parameters generated by each layer of the model is termed the Number of Parameters Generated by the Model (NPM). The size of the final trained model is called Model Size(S). The mathematical form of the metrics are shown in Eqs. (19)–(26).

19 $$\:\mathrm{A}\:=\:\frac{\mathrm{T}\mathrm{P}+\mathrm{T}\mathrm{N}}{\mathrm{T}\mathrm{P}+\mathrm{F}\mathrm{P}+\mathrm{T}\mathrm{N}+\mathrm{F}\mathrm{N}}$$

20 $$\:\mathrm{L}\:=\:-\sum\:_{\mathrm{i}=1}^{\mathrm{N}}\sum\:_{\mathrm{j}=1}^{\mathrm{M}}{\mathrm{A}}_{\mathrm{i}\mathrm{j}}\times\:\mathrm{log}{\mathrm{P}}_{\mathrm{i}\mathrm{j}}$$

21 $$\:\mathrm{P}=\frac{\mathrm{T}\mathrm{P}}{\mathrm{T}\mathrm{P}+\mathrm{F}\mathrm{P}}$$

22 $$\:\mathrm{R}=\frac{\mathrm{T}\mathrm{P}}{\mathrm{T}\mathrm{P}+\mathrm{F}\mathrm{N}}$$

23 $$\:\mathrm{F}\mathrm{S}=2\times\:\:\frac{P\times\:R}{P+R}$$

24 $$\:MIoU=\:\frac{1}{N}\sum\:_{i=1}^{N}\frac{{L}_{i}\:\cap\:\:{PL}_{i}}{{L}_{i}\:\cup\:\:{PL}_{i}}$$

25 $$\:\mathrm{M}\mathrm{R}\mathrm{T}=\frac{\sum\:_{i=1}^{N}{RT}_{i}}{N}$$

26 $$\:\mathrm{N}\mathrm{P}\mathrm{M}=\sum\:_{i}^{L}{NP}_{i}$$

where, TP – True Positive, FP - False Positive, TN - True Negative, FN - False Negative, P – Precision, R – Recall, $\:{RT}_{i}$ – Response Time for segmenting i^th^ crop field image, $\:{NP}_{i}$ – Number of parameters generated by the i^th^ layer, $\:{P}_{ij}$ – The predicted pixel value by the model, $\:{A}_{ij}$ – The actual pixel value in the target label, N – Total number of samples, M - Total number of classes, L – Total number of layers present in the model.

## Results and discussion

The evaluation and comparison of the performance of the proposed model are done in four different categories, which are.

1. Performance comparison of the proposed model on multi-weed segmentation.
2. Performance comparison between the proposed model vs. existing state-of-the-art works.
3. Ablation analysis for component-wise evaluation of the proposed model.
4. Evaluating the suitability of the proposed model on Mobile/Edge devices.

### Performance comparison of the proposed model on multi-weed segmentation

This study uses deep learning models like SegNet512^7^, U-Net, RRUDC, UNet++, and DeepLabV3+^10^ to evaluate the effectiveness of the proposed CAUC model in multi-weed segmentation. All of these models, including the proposed CAUC model, are fine-tuned using the training dataset (Table 1) in the experimental settings outlined in Sect. 4.1. First, Accuracy (A), Loss (L), Precision (P), Recall (R), F1-Score (FS), and Mean Intersection over Union (MIoU) are used to assess how well the model segments crop and weed regions. Table 4 documents the corresponding outcomes from the test and validation stages.

The comparison results depicted in Table 4 indicate the efficiency of the proposed CAUC model and also provide insight into the performance of existing state-of-the-art segmentation models. The CAUC model achieved 99.09% validation set and 97.50% test set accuracy with good generalizability over models such as SegNet and RRUDC that achieved relatively lower accuracies for both stages. Keeping the loss also in view, CAUC attained 2.77% for validation and 7.85% for testing, which puts it at a competitive standing amongst other state-of-the-art architectures. In addition, the proposed model achieved Precision and Recall of 99.11% and 99.02% on the validation set and 97.14% and 96.90% on the test set, respectively, which resulted in the highest F-scores of all models (99.06% and 97.02%).

Although SegNet and RRUDC obtained reasonable validation results, their relatively low test recall and precision levels suggest lower consistency for hard segmentation problems. UNet and UNet + + competed vigorously, with UNet + + offering the best validation accuracy of 99.22% and stable consistency across the metrics. DeepLabV3 + also attained stable consistency levels of accuracy with 99.02% on validation and 97.09% on test, demonstrating its good baseline potential for semantic segmentation. Further, the best aspect of the CAUC model remains its Mean Intersection over Union (MIoU), which achieved 81.02% on validation and 80.20% on test. These metric scores are slightly lower than the UNet + + and DeepLabV + + models’ metric scores. As a primary quality measure of segmentation tasks, the outcome demonstrates the strength of the CAUC model at discerning thinner borders of weeds from crops by a greater margin, thereby confirming its robustness potential for realistic application cases of multi-crop field crop and weed image segmentation.

**Table 4:** The comparison of outcomes attained by the proposed CAUC model and other state-of-the-art models in the Multi-weed segmentation.

Model name	Validation time						Test time					
	A	L	*P*	*R*	FS	MIoU	A	L	*P*	*R*	FS	MIoU
	%	%	%	%	%	%	%	%	%	%	%	%
SegNet512[23]	97.46	6.25	97.62	97.32	97.46	78.42	95.81	18.69	95.98	95.68	95.82	75.23
UNet [7]	98.85	4.18	98.88	98.82	98.84	80.05	96.89	19.02	97.01	96.79	96.89	78.52
RRUDC [7]	97.76	4.62	97.78	97.74	97.76	79.52	95.92	8.52	96.01	95.84	95.92	77.25
DeepLabV3+	99.02	3.65	99.06	98.99	99.02	81.11	97.09	5.6	97.27	96.97	97.11	80.61
UNet++	99.22	2.5	99.25	99.20	99.22	81.55	97.35	5.7	97.41	97.30	97.35	80.85
**Proposed CAUC**	**99.09**	**2.77**	**99.11**	**99.02**	**99.06**	**81.02**	**97.50**	**7.85**	**97.14**	**96.9**	**97.02**	**80.2**

**Fig. 10:**
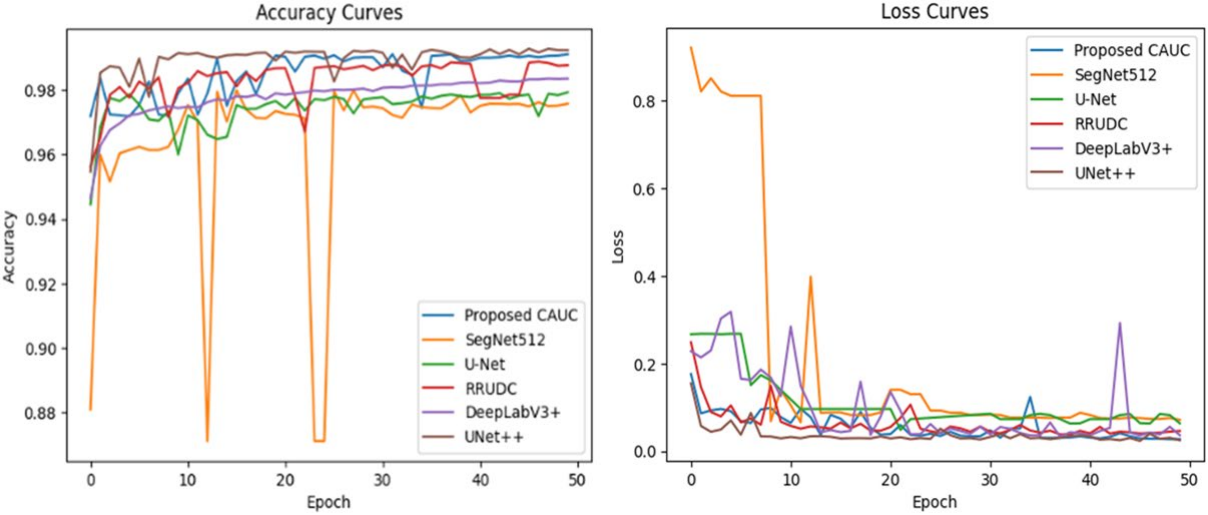
The validation time- Accuracy and Loss curves of the proposed CAUC, SegNet512, UNet, RRUDC, DeepLabV3+, and UNet + + models over the training epochs. (**a**) Accuracy curves, (**b**) Loss curves.

**Fig. 11:**
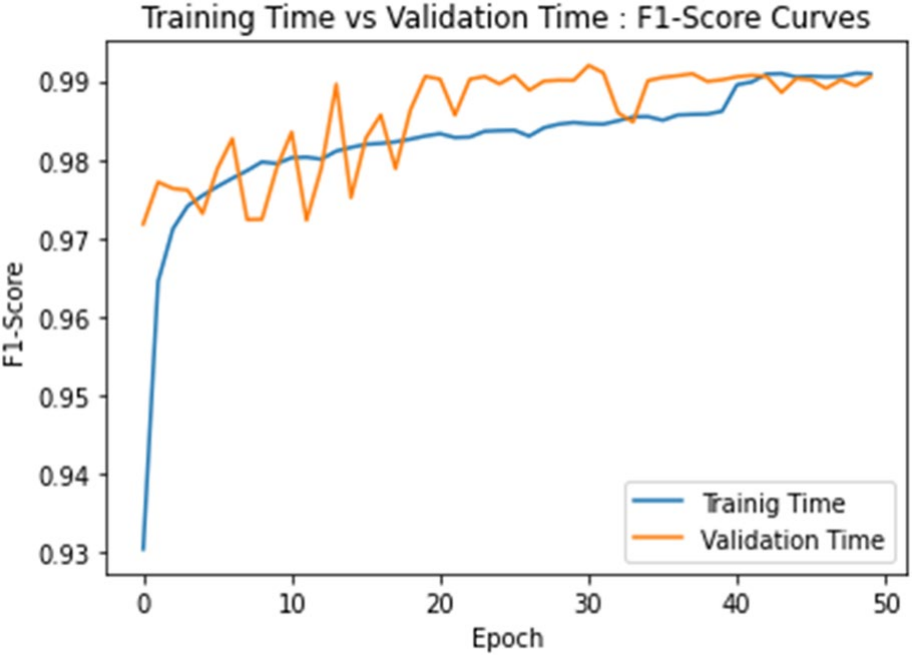
The F1- Score curves of the proposed CAUC model in training and validation time.

The proposed CAUC and state-of-the-art models’ validation time accuracy, and Loss curves are visualized in Fig. 10. With relatively small variation in the learning curves, the CAUC model achieves consistently higher accuracy, which proves stable convergence. SegNet512 exhibits instability with sharp descents. UNet + + and DeepLabV3 + rival but show occasional variation, while U-Net and RRUDC progress continuously with moderate variation. In terms of loss, CAUC achieves a smooth decline and converges at lower levels than most models, confirming its optimization efficiency. SegNet512 shows the instability with large spikes, while other models converge moderately well but at higher loss levels. Additionally, the proposed CAUC model’s epoch-wise F1-Scores learning curves of the training and validation stages are plotted in Fig. 11. From Figs. 10 and 11, one can observe that the learning curves of Accuracy, Loss, and F1-Score for the CAUC model proposed are converging and trending towards higher values, post the 40 epochs.

From Fig. 10a, all models’ accuracy learning curves begin from ~ 94% because soil portions in every image are much higher than the vegetation portions. Hence, calculating overall accuracy may not be the right choice to evaluate the preciseness of crop and weed segmentations perfectly, since the segmentation of soil portions dominates the overall accuracy. Accordingly, Precision, Recall, and FS scores are calculated independently for Carrot, Sunflower, Sugar beet, and weed crops, and Table 6 presents the corresponding results. According to Table 5, the FS score of the proposed CAUC model for the vegetation is more than 90% in both validation and test time, besides weed portions, where the FS scores on segmenting weed portions will be approximately 82% to 87%. Due to the portions occupied by the weed being smaller in size when compared to crop portions, a drop might be observed in the proposed model.

**Table 5:** The comparison of the proposed model’s performance in segmenting the vegetation portions.

S. no	Vegetation	Validation time			Test time		
		*P* (%)	*R* (%)	FS (%)	*P* (%)	*R* (%)	FS (%)
1	Carrot	93.66	92.59	93.12	91.27	90.59	90.93
2	Sugar beet	97.51	97.61	97.56	92.51	91.61	92.06
3	Sunflower	96.26	95.46	95.86	90.25	97.79	93.87
4	Weed	87.95	83.87	85.86	84.95	82.37	83.64

The applications are designed to create light, quick-reacting models that not only improve performance but are also deployable on resource-limited devices to address farmers’ requirements. For this purpose, all models are compared based on Model Size, Number of Parameters (NPM), and Mean Runtime (MRT), and the respective values are tabulated in Table 6.

**Table 6:** The comparison of model size (S), number of parameters generated by the model (NPM), and mean response time (MRT).

S. no	Model name	S	NPM	MRT
		MB	Million	seconds
1	SegNet512^7^	185.35	16.35	1.53
2	U-Net^10^	63.5	5.51	0.62
3	RRUDC^10^	7.9	0.65	0.30
4	DeepLabV3+^50^	153.8	13.39	1.51
5	UNet++^51^	104	9.05	1.07
6	Proposed CAUC	5.6	0.37	0.35

*S* model size, *NPM* number of parameters generated by the model, *MRT* mean response time.

Table 6 compares model size, the number of model parameters, and the Mean Response Time(MRT) of the model, which indicates the efficiency of model computation. Although state-of-the-art semantic segmentation models, namely SegNet512 and DeepLabV3+, are extremely large (185.35 MB and 153.8 MB models, respectively) and require model parameters (16.35 M and 13.39 M), the CAUC model is remarkably small. Because the CAUC model is only 5.6 MB with only 0.37 million computational parameters. Compared by MRT, the CAUC model (0.35 s) beats large models such as SegNet512 and DeepLabV3 + at the speed of inference but matches the extremely light RRUDC model at 0.30 s. Compared with U-Net and UNet++, the CAUC model also consumes fewer parameters and occupies less memory while maintaining a competitive response time.

Further, the computational efficiency vs. performance trade-off is analyzed by comparing the outcomes of Tables 4 and 6. The CAUC model achieves slightly higher validation (2.77%) and testing (7.85%) loss values than those of UNet++ (2.5% validation, 5.7% testing) and DeepLabV3+ (3.65% validation, 5.6% testing). However, CAUC achieves steadily high segmentation quality, an MIoU of 81.02% on validation data and of 80.20% on test data. These values stand their ground against the best-performing UNet++ (81.55% validation, 80.85% testing) and DeepLabV3+ (81.11% validation, 80.61% testing). Moreover, CAUC achieves stable levels of accuracy (99.09% validation, 97.50% testing) and F-scores (99.06% validation, 97.02% testing) superior even to large SegNet512 and RRUDC models. Although SegNet512 and DeepLabV3 + deploy significantly higher computing resources, they fail to bring corresponding performance gains. This indicates that CAUC achieves an optimal balance, embedding compactness, fast response time, and high segmentation quality appropriate for deployment at large scales in real-time agriculture.

**Fig. 12:**
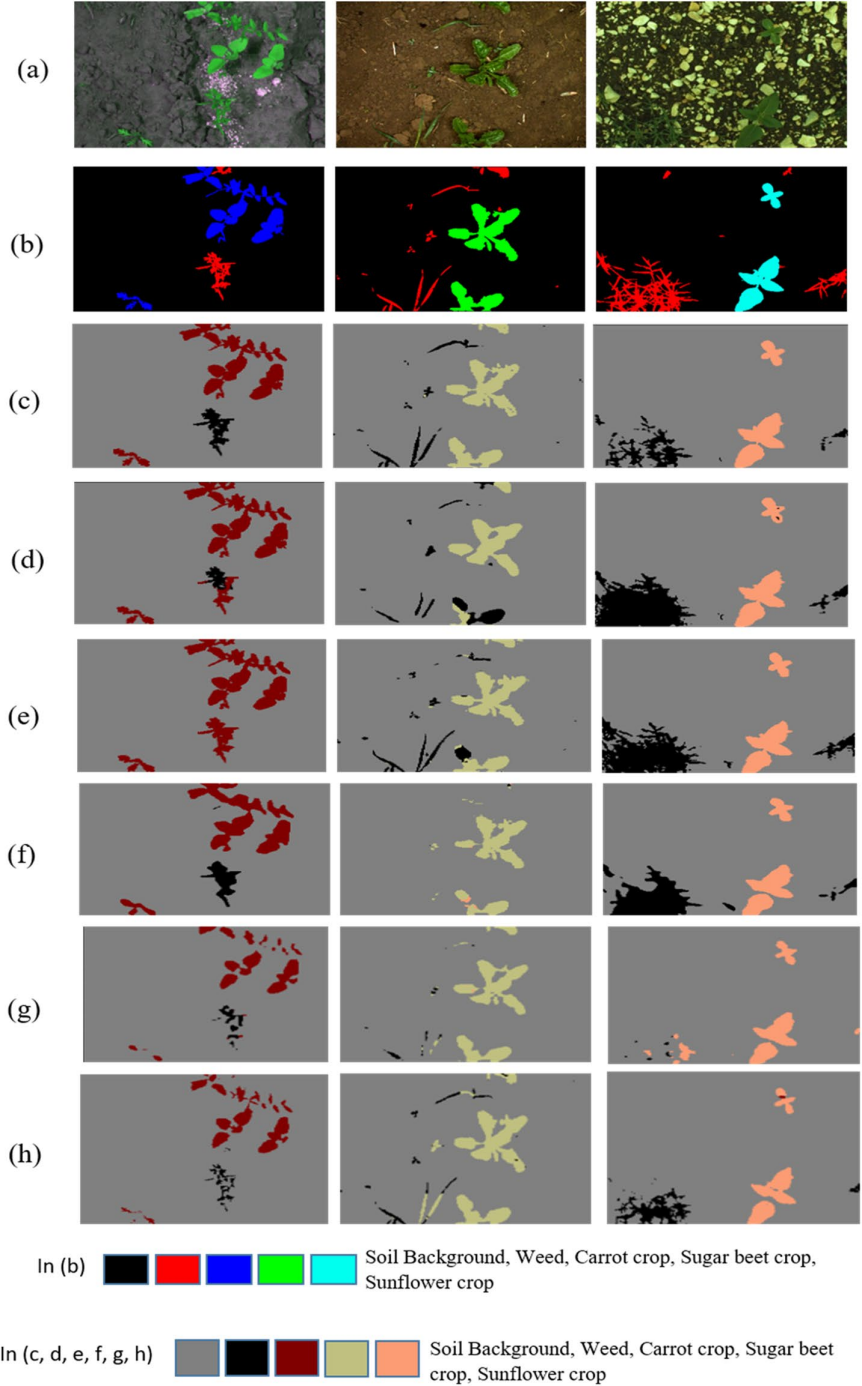
Crop and weed portions segmented image for the sample image from Carrot Crop (CWFID), Sugar Beet Crop, and Sunflower Crop dataset, generated by different Models, (**a**) Actual crop field images, (**b**) Actual target label images, (**c**) Segmented images generated by the Proposed CAUC model, (**d**) Segmented images generated by the RRUDC model, (**e**) Segmented images generated by the UNet model, (**f**) Segmented images generated by the SegNet512 model, (**g**) Segmented images generated by the DeepLabV3+, (**h**) Segmented images generated by the UNet++.

Figure 12 shows the qualitative comparison of crop and weed segmented images generated by the models, such as the proposed CAUC model, RRUDC, UNet++, SegNet512, DeepLabV3+, and U-Net for the sample image from the Carrot Crop (CWFID), Sugar Beet Crop, and Sunflower Crop datasets. The actual crop field images and their ground truth are given in Fig. 12a,b, respectively. Generated Segmented images from the proposed CAUC, RRUDC, UNet++, SegNet512, DeepLabV3+, and U-Net are shown in Fig. 12c–h, respectively. Visualization shows that the proposed CAUC model (Fig. 12c) provides a precise crop and weed segmented image that is more aligned with the actual target label image (Fig. 12b).

Compared with other state-of-the-art semantic segmentation models’ crop and weed segmented images, the proposed model’s crop and weed segmented images have finer crop boundaries, correct separation of weed from crop area, and reduced misclassification in high vegetation density. The architectural components of the proposed CAUC, such as AG-attached skip connections among the corresponding encoder and decoder blocks, and integration of CBAM in the decoder, enable more precise crop and weed segmentation with precise boundaries. Next to this, the UNet + + model achieved a precise crop and weed segmentation and reduced misclassifications due to nested skip connections (Fig. 12.h). However, it may slightly oversmooth fine weed structures, resulting in minor detail loss compared to the ground truth.

Although SegNet512 and DeepLabV3+ (Fig. 12f, g) over-segment or blur crop-weed interfaces, and lightweight networks like RRUDC (Fig. 12d) sometimes lose distinct structures. Furthermore, the proposed CAUC model attained a trade-off between semantic segmentation performance and computational complexity. This proves qualitatively the quantitative enhancements indicated in the results of Tables 4 and 6, affirming the CAUC model’s robustness in multi-weed segmentation.

### Performance comparison between the proposed model vs. existing state-of-the-art works

Comparing the performance of the proposed model with existing state-of-the-art approaches is one of the crucial steps in assessing the proposed model’s efficiency. However, the existing state-of-the-art crop and weed segmentation approaches are applied to a single crop field. Although few existing works processed multiple crop field images in their research work, they have not used these three crops (carrot, sugar beet, sunflower) together in their research work. Hence, to evaluate how the proposed CAUC model is more efficient in terms of crop and weed segmentations, and computationally effective than the existing state-of-the-art crop and weed segmentation approaches, the proposed CAUC model is trained using the individual datasets of carrot, sugar beet, and sunflower crops, and their outcomes are compared with their corresponding existing work results, presented in Table 7.

Based on the inference recorded in Table 7, the computational complexity level of the proposed CAUC model in terms of NPM is relatively low compared to other models except for the AgNet model^9^, where the AgNet model achieved crop and weed segmentation accuracy on the carrot crop that is less (88.9%) when compared to the proposed CAUC model (98.38%). Regarding Recall, the RRUDC model^10^ achieved a better score (98.82%) when compared to the proposed CAUC model (97.51%), but the difference is in the negligible range. However, the proposed CAUC model minimizes 42% of the computational complexity as compared to the RRUDC model. The crop and weed segmentation accuracy of the Bonnet model^26^ on Sugar beet crops is a little high (99.32%) but in a negligible range in comparison with the proposed CAUC model. Despite this, the parameter reduction on the proposed CAUC model is 34% when compared to the Bonnet model^26,52^. According to the above discussion, the proposed CAUC model outperformed existing deep learning-based models/approaches based on the trade-off between performance and computational complexity.

**Table 7:** The performance and complexity comparison between the proposed CACU model vs state-of-the-art approaches.

Crop	Model/approach name	A	MIoU	P	R	NPM
		%	%	%	%	Million
Carrot	DeepLabV3++^28^	84.3	–	–	–	11.85
	Adapted-IV3^9^	93.9	–	–	–	25
	AgNet^9^	88.9		–	–	0.25
	RRUDC^10^	95.40	–	95.43	98.82	0.655
	Proposed CAUC	98.38	80.5	97.51	97.27	0.377
Sugar Beet	Deep encoder-decoder CNN(Bonn)^53^	94.74	80.1	–	–	–
	UNet-ResNet50(dice + focal)^29^	96.06	85.25	92.28	92.21	20.67
	Bonnet^26^	99.32	77.47	–	–	1.1
	UNet-ResNet50^21^	–	67.0	–	–	20.67
	Proposed CAUC	98.51	80.75	97.54	97.50	0.377
Sun flower	VGG-UNet^31^	90.0	64.0	–	–	–
	Bonnet^21^	–	70.0			1.1
	UNet-ResNet50^21^	–	43.0			20.67
	Bonnet^26^	99.02	68.98	–	–	1.1
	Proposed CAUC	99.10	81.1	99.14	99.10	0.377

A - Accuracy, P – Precision, R - Recall, S – Model Size, NPM - Number of Parameters generated by the Model, - Not given in the paper.

### Ablation analysis for component-wise evaluation of the proposed model

A component-wise ablation analysis of the proposed model is a valuable analytical tool in deep learning-based research, assessing the significance and contribution of individual components within the model. The purpose of this study is to isolate specific components from the proposed CAUC model in different ways and observe their effects on model performance. It enables a better understanding of what components drive the model’s precise crop and weed segmentation. In this regard, the proposed CACU model is created as 4 versions by removing AG, CBAM, and LCB components in different ways, which are CAUC without AG (CAUC-wo-AG), CAUC without CBAM (CAUC-wo-CBAM), CAUC without AG and CBAM (CAUC-wo-CBAM_AG), CAUC without LCB, AG, and CBAM (CAUC-wo-CBAM_AG_LCB).

CAUC-wo-AG (V3) uses a traditional skip connection in place of the AG-attached skip connection. Whereas CAUC-wo-CBAM (V4) derives the final output from the last LCB component and removes only the CBAM, CAUC-wo-CBAM_AG (V2) excludes both the AG and CBAM components. Finally, CAUC-wo-CBAM_AG_LCB removes the AG, CBAM, and LCB components and operates similarly to a typical UNet architecture. All these ablated versions of proposed models, CAUC-wo-CBAM_AG_LCB (V1), CAUC-wo-CBAM_AG (V2), CAUC-wo-AG (V3), and CAUC-wo-CBAM (V4), are built and trained in the same execution environment, and the corresponding outcome is recorded in Table 8.

**Table 8:** Comparison of results attained in the ablation study on the proposed CAUC model.

Ver. no	Model name	Size	NPM	Validation time		Test time		Observation	
				FS	MIoU	FS	MIoU	Performance	Computational complexity
		MB	Million	(%)	(%)	(%)	(%)		
V1	CAUC-wo-CBAM_AG_LCB	63.4	5.51	98.84	80.05	96.89	78.52	High	Very High
V2	CAUC-wo-CBAM_AG	4.1	0.281	96.27	75.26	94.74	73.12	Low	~ 93.53% less than V1
V3	CAUC-wo-AG	4.3	0.288	97.70	79.11	95.82	77.55	Improved over V2 Less than V1	Slightly increased from V2
V4	CAUC-wo-CBAM	5.26	0.370	97.69	79.21	95.74	77.65	Improved over V2 Less than V1	Slightly increased from V2 & V3
V5	Actual proposed CAUC model	5.6	0.377	99.05	81.02	96.92	80.2	Higher than all	Slightly increased from V3 & V4.

The outcome of the Ablation study presented in Table 8 not only confirms individual contributions of LCB, AG, and CBAM but also reflects their complementary interactions and joint effect on performance. When all three modules are excluded (CAUC-wo-CBAM_AG_LCB, V1), the model achieved relatively high FS and MIoU (98.84% and 80.05%), but with the cost of very high computational complexity (63.4 MB, 5.51 M parameters), revealing that the backbone is capable of extracting features but is not computationally effective. Meanwhile, excluding AG and CBAM (CAUC-wo-CBAM_AG, V2) resulted in the greatest decline of performance (FS: 96.27%, MIoU: 75.26), revealing their significant contribution to fine-tuning contextual and attention-related feature representations, but such a variant had the largest number of parameter reduction (~ 93.5% fewer compared with V1). Removing AG alone (CAUC-wo-AG, V3) resulted in better performance compared with V2 (FS: 97.70%, MIoU: 79.11), revealing that LCB improves localized context coding even without AG. Likewise, excluding CBAM alone (CAUC-wo-CBAM, V4) resulted in results close to V3 (FS: 97.69%, MIoU: 79.21%), implying AG and CBAM play complementary but partially intersecting contributions, with AG enhancing scale-crossed spatial dependency and CBAM highlighting channel-spatial discriminative features. The entire CAUC model (V5) with LCB, AG, and CBAM correlated showed the best performance (FS: 99.05%, MIoU: 81.02) with a slight increase in computational complexity compared with (CAUC-wo-AG, V3) and (CAUC-wo-CBAM, V4), but verifies that the three modules synergistically interact with each other. Precisely, LCB enriches localized feature aggregations, AG refines multi-scale context information, and CBAM focuses on attention on the most discriminative area with better accuracy while remaining efficient.

Based on Tables 4, 5, 6, 7 and 8, the proposed CAUC model satisfies requirements, such as a better-performing model with lesser computational complexity and model size, when building agricultural computer vision applications. It has achieved the highest Accuracy, MIoU, and F1-scores of 97.5%, 80.2%, and 97.02% in test time with the lowest Model size, NPM of 5.6 MB, and 0.37 million. It also consumes the second-lowest MRT of 0.35 s.

### Evaluating the suitability of the proposed model on mobile devices

To estimate the suitability and the performance of the developed CAUC model on low-computational devices, a computer vision application has been developed using the proposed CAUC model for deployment on mobile devices. The trained proposed CAUC model in .h5 format is transformed into TensorFlow Lite format to accomplish this. During this conversion, the size of the proposed CAUC model is reduced from 5.6 MB to 1.5 MB. Hence, the developed computer vision application for crop and weed segmentation comes in a lightweight 13.7 MB size whose memory consumption on the mobile device is shown in Fig. 9. This developed computer vision application is deployed on three mobile devices of different configurations to identify the response time on a range of mobile devices. The MRT is calculated on these three mobile devices using the Test dataset images, and their corresponding values are recorded in Table 9.

**Table 9:** Comparison of MRT for the developed computer vision applications on different configuration mobile devices.

Mobile device	Configuration	MRT
		seconds
MD 1	RAM: 1 GB, Storage: 16 GB, Quad-Core, Android 6	2.4
MD 2	RAM: 3GB, Storage: 32 GB, Octa Core, Android 11	1.3
MD 3	RAM: 4GB, Storage: 32 GB, Octa Core, Android 11	0.4

**Fig. 13:**
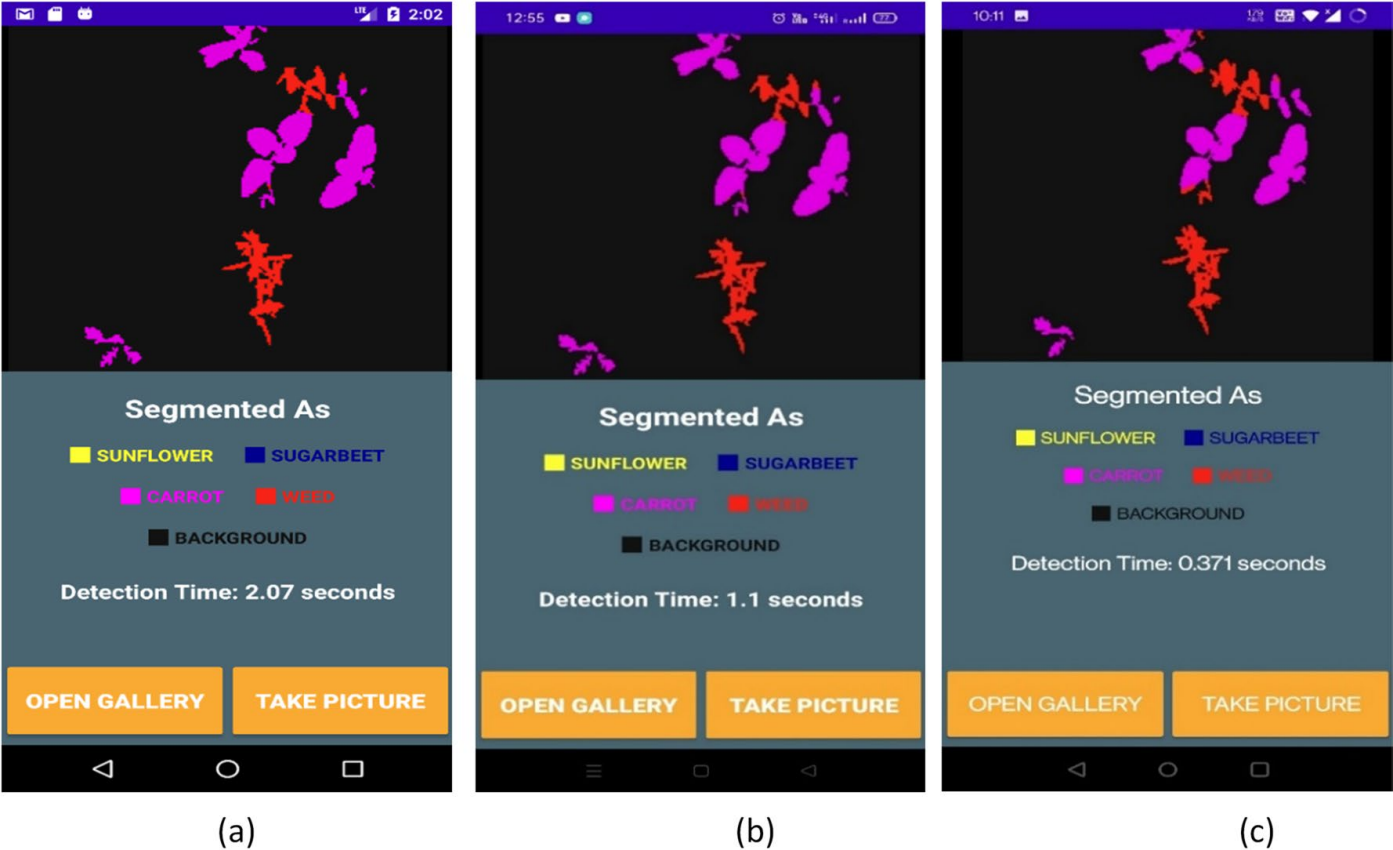
The comparison of detection time consumed for crop and weed segmentation done on three different configuration mobile devices. (**a**) On Mobile Device - MD1, (**b**) On Mobile Device – MD2, (**c**) On Mobile Device – MD3.

A crop-field sample image is segmented by the recommended computer vision mobile app on various mobile devices, as indicated in Fig. 13. The reference segmented image produced by the CAUC model using Colab is depicted in Fig. 12.c(1). Even with differences in detection time on various mobile device setups, the weed and crop segmentation is still accurate, as presented in Fig. 13.

## Conclusion

A lightweight Concatenated Attention U-Net with CBAM (CAUC) is proposed in this research work to segment the crop and weed portions in the three agricultural crop fields (carrot, sugar beet, and sunflower). The core components of the proposed CAUC model are Linear Concatenated Block (LCB), Attention Gate (AG), and Convolutional Block Attention Module (CBAM). A key role played by the components AG and CBAM is to utilize the feature maps efficiently and help the flow of significant features among LCB components without increasing computational complexity excessively.

The design of LCB components is such a way that it reduces the computational complexity using the two approaches without degrading the performance of the model. With the depth-wise separable convolution layers in the first approach, the computational parameter reduction per LCB component (PR1) is achieved in the range of ~ 12% to ~ 17%. Using 1 × 1 convolution layers, the second approach achieves computational parameter reductions of 63.5% and 52.5% on the encoder and decoder sides, respectively.

With this design strategy, the proposed CAUC model achieved better performance in segmenting the crop and weed portions with an Accuracy and F1-score of 97.50% & 97.02% with the computational complexity, such as the model size of 5.6 MB and 0.377 million computational parameters, and the MRT of 0.35 s/image. A lightweight computer vision application of size 13.7 MB is developed to aid the farmers using this proposed model, and the same is deployed on different mobile devices for performance evaluation, where the performance of crop and weed segmentation is not affected in different computational devices with slight variations in the MRT.

## Limitations and future work

- To assess the suitability of our proposed model in resource-constrained devices, we built a computer vision-based mobile application using the proposed model and tested different configurations of mobile devices. However, the model is not tested under edge devices in the real agricultural fields, and the real-time deployment, which will be carried out in our future work.
- The integration of a new category of crop field images into the existing model will demand retraining of the model after the inclusion of the new crop field images, which will be carried out in future work.
- The computational complexity is optimized further using Knowledge-Distillation techniques in future work.

## Data Availability

The data that support the findings of this study are openly available in [Dataset Ninja, Image Synthesis] at https://datasetninja.com/cwfid, https://datasetninja.com/sugar-beets-2016, https://sites.google.com/diag.uniroma1.it/image-synthesis/downloads?authuser=0, reference numbers^27,38,37^.
